# Left Atrial Appendage Thrombus and Long-Term Clinical Outcomes in Anticoagulated Patients with Atrial Fibrillation and Atrial Flutter Referred for a Rhythm-Control Strategy: A Prospective Observational Cohort Study

**DOI:** 10.3390/jcm15218552

**Published:** 2026-11-03

**Authors:** Łukasz Turek, Marcin Sadowski, Łukasz Zandecki, Jacek Kurzawski, Marianna Janion

**Affiliations:** Faculty of Medicine, Jan Kochanowski University in Kielce, al. IX Wieków Kielc 19A, 25-516 Kielce, Poland

**Keywords:** atrial fibrillation, atrial flutter, cardiovascular death, heart failure, left atrial appendage thrombus, oral anticoagulation, rhythm control

## Abstract

**Background/Objectives**: Whether long-term prognosis differs between patients with atrial fibrillation (AF) and those with atrial flutter (AFL) after adjustment for baseline cardiovascular characteristics remains unclear. **Methods**: In this prospective observational cohort study, we enrolled 656 patients meeting predefined anticoagulation eligibility criteria with AF (*n* = 558) or AFL (*n* = 98) who underwent transesophageal echocardiography (TEE) before a planned rhythm-control intervention. The primary endpoint was left atrial appendage thrombus (LAAT) detected at the index TEE. Long-term outcomes included cardiovascular death, heart failure (HF) hospitalization, ischemic stroke, transient ischemic attack, myocardial infarction, and systemic thromboembolism. **Results**: LAAT was detected in 14.2% of patients with AF and 10.2% of patients with AFL (*p* = 0.29). After multivariable adjustment, AFL versus AF was not independently associated with LAAT (adjusted odds ratio [aOR], 0.53; 95% confidence interval [CI], 0.25–1.11; *p* = 0.09). Median follow-up was 6.85 (3.64–8.53) years. Cardiovascular death occurred in 96 patients (14.6%; AF: *n* = 78, AFL: *n* = 18), and HF hospitalization occurred in 91 patients (13.9%; AF: *n* = 78, AFL: *n* = 13). Log-rank comparisons were not statistically significant for cardiovascular death (log-rank *p* = 0.42) or HF hospitalization (log-rank *p* = 0.73). In extended Cox models that accounted for nonproportional hazards, AFL versus AF was not independently associated with cardiovascular death (adjusted hazard ratio [aHR], 1.09; 95% CI, 0.64–1.85; *p* = 0.75) or HF hospitalization (aHR, 0.69; 95% CI, 0.37–1.27; *p* = 0.23). **Conclusions**: Catheter ablation was recorded in 83.7% of AFL versus 24.6% of AF patients, including index-hospitalization ablation in 46.9% versus 0%, respectively. In this selected cohort meeting predefined anticoagulation eligibility criteria referred for TEE-guided rhythm control, no statistically significant independent association was detected between index arrhythmia type (AF versus AFL) and LAAT, cardiovascular death, or HF hospitalization. Importantly, these findings describe associations under markedly different subsequent rhythm-control treatment pathways and do not establish equivalent intrinsic risks for AF and AFL. The smaller AFL group (*n* = 98) and wide confidence intervals limit precision; clinically meaningful differences cannot be excluded. These findings should not be extrapolated to unselected AF/AFL populations, including asymptomatic patients, those managed exclusively with rate control, or patients not receiving anticoagulation. The comparison is based on presenting arrhythmia, not a rhythm phenotype confirmed to persist throughout follow-up; incident AF among patients initially presenting with AFL was not systematically ascertained.

## 1. Introduction

Atrial fibrillation (AF) and atrial flutter (AFL) are the two most common sustained atrial tachyarrhythmias encountered in contemporary clinical practice [1]. Although they differ in their electrophysiological mechanisms [1,2], both arrhythmias are associated with increased risks of thromboembolism, heart failure (HF), and cardiovascular death [3,4]. Consequently, contemporary management of both AF and AFL includes comprehensive cardiovascular risk reduction, oral anticoagulation when indicated, and rhythm-control strategies tailored to each patient [1,2].

Despite these similarities, rhythm-control approaches differ between AF and AFL. While both arrhythmias may be managed with antiarrhythmic drugs, electrical cardioversion, and catheter ablation depending on the clinical setting, catheter ablation has a particularly well-established role in the treatment of typical cavotricuspid isthmus-dependent AFL [2]. In contrast, management of AF is more individualized and may involve one or more of these approaches, depending on patient characteristics, symptom burden, and shared decision-making [1]. These differences in management are relevant when comparing clinical outcomes between the two groups.

Previous studies have predominantly reported outcomes for AF and AFL as a combined population [5,6], whereas direct comparisons between AF and AFL as separate clinical entities remain relatively uncommon [4,7,8,9]. Therefore, it remains uncertain whether index arrhythmia type is independently associated with long-term clinical outcomes after adjustment for baseline clinical characteristics.

To directly compare AF and AFL within a common analytical framework, we conducted a prospective observational cohort study of consecutive adequately anticoagulated patients with AF or AFL referred for transesophageal echocardiography (TEE) before a planned rhythm-control strategy. Baseline clinical characteristics, echocardiographic findings, and treatment data were prospectively collected, and patients were followed for predefined clinical outcomes. We hypothesized that index arrhythmia type would not be independently associated with long-term clinical outcomes after adjustment for baseline clinical characteristics.

Here, anticoagulation eligibility was defined by serial international normalized ratio (INR) testing for vitamin K antagonist (VKA) users and by patient-reported uninterrupted use for direct oral anticoagulant (DOAC) users, rather than by objectively verified adherence across all regimens.

## 2. Materials and Methods

### 2.1. Study Population

This prospective observational cohort study was conducted at the Provincial Combined Hospital in Kielce, Poland, using data collected from its electronic medical database. Consecutive adult patients meeting predefined oral anticoagulation eligibility criteria who were admitted to the Department of Cardiology for TEE as part of a planned rhythm-control strategy for AF or AFL were prospectively enrolled. Patients with AF were recruited between 13 December 2010 and 22 April 2024, whereas patients with AFL were recruited between 31 January 2011 and 4 April 2024. The date of the index TEE defined study enrollment and the start of follow-up. Enrollment ended in April 2024 to allow at least 12 months of potential follow-up through 30 April 2025; follow-up could end earlier because of death. The inclusion and exclusion criteria are presented in Table 1.

TEE was performed in all participants before the planned rhythm-control intervention. Following completion of the diagnostic work-up, the treating physician selected the management approach (direct-current cardioversion, catheter ablation, or conservative management) based on the patient’s clinical presentation, TEE findings, and contemporary guideline recommendations.

When left atrial appendage thrombus (LAAT) was detected, the planned rhythm-control intervention was postponed and oral anticoagulation was continued in all affected patients. The anticoagulant agent was changed in some patients, whereas others remained on their existing treatment, at the discretion of the treating physician.

The date of the index TEE was defined as the baseline (index date) and marked the start of follow-up for all time-to-event analyses. Baseline clinical data, including demographic characteristics, medical history, laboratory findings, pharmacological treatment, and echocardiographic parameters, were collected during the index hospitalization.

A history of HF was defined as a previously documented clinical diagnosis of heart failure in the patient’s medical records before the index hospitalization, irrespective of left ventricular ejection fraction (LVEF) measured at the index echocardiographic examination.

Patients were categorized as having AF or AFL according to the arrhythmia documented at the index evaluation. Systematic electrocardiographic or ambulatory rhythm monitoring was not performed during follow-up; therefore, subsequent AF occurrence in the AFL group and longitudinal atrial arrhythmia burden were not assessed as study variables.

All 98 patients in the AFL group were classified by the investigators as having typical atrial flutter based on the index electrocardiographic findings. In patients who underwent catheter ablation, this classification was additionally supported by findings obtained during the electrophysiological procedure. Electrophysiological confirmation of cavotricuspid isthmus dependence was not available for all patients.

The study was conducted in accordance with the ethical principles of the Declaration of Helsinki. Ethical approval was obtained from the Ethics Committee of the Jan Kochanowski University of Humanities and Sciences in Kielce, Poland (protocol code: 21/2010; approved on 28 January 2010). Informed consent was obtained from all participants before study enrollment. This study is reported in accordance with the Strengthening the Reporting of Observational Studies in Epidemiology (STROBE) statement. The study was not registered in a public study registry.

### 2.2. Oral Anticoagulation

Meeting predefined oral anticoagulation eligibility criteria was mandatory for study enrollment. Before TEE, all participants were required to meet one of the following anticoagulation criteria: treatment with VKA with dose adjustment and INR monitoring to maintain therapeutic anticoagulation (INR ≥ 2.0), or uninterrupted treatment with apixaban, rivaroxaban, or dabigatran for at least 3 weeks before the planned rhythm-control intervention [10,11,12,13]. For participants treated with VKAs, three consecutive weekly INR measurements were required during the 3 weeks preceding enrollment, with each measurement ≥ 2.0. For participants receiving direct oral anticoagulants, uninterrupted treatment during the preceding 3 weeks was assessed solely on the basis of patient declarations; no objective measure of adherence was available. Accordingly, “adequate anticoagulation” in this study denotes fulfillment of the prespecified eligibility criteria and should not be interpreted as objectively verified DOAC adherence or therapeutic drug exposure in every participant. No upper INR threshold was specified as a study eligibility criterion.

For the purpose of study eligibility, the standard doses were dabigatran 150 mg twice daily, rivaroxaban 20 mg once daily, and apixaban 5 mg twice daily. Reduced-dose regimens were also considered acceptable for study inclusion when predefined dose-reduction criteria were met. These included dabigatran 110 mg twice daily in patients with at least one of the following: concomitant verapamil treatment, age ≥ 80 years, a HAS-BLED score ≥ 3, or estimated glomerular filtration rate (eGFR) 30–49 mL/min/1.73 m^2^; rivaroxaban 15 mg once daily in patients with a HAS-BLED score ≥ 3 or eGFR 15–49 mL/min/1.73 m^2^ [11]; and apixaban 2.5 mg twice daily in patients meeting at least two of the following: serum creatinine ≥ 1.5 mg/dL, body weight ≤ 60 kg, or age ≥ 80 years. Renal function was evaluated using eGFR calculated with the Modification of Diet in Renal Disease (MDRD) equation [14].

These predefined study eligibility criteria reflected historical European Society of Cardiology (ESC) recommendations applicable during the recruitment period and should be distinguished from regulatory product-label dose-reduction criteria. Importantly, a HAS-BLED score ≥ 3 alone is not a labelled criterion for reducing the rivaroxaban dose from 20 to 15 mg once daily. For dabigatran, the product information permits consideration of a reduced dose of 110 mg twice daily in selected patients at increased bleeding risk following an individualized benefit–risk assessment, but does not specify a HAS-BLED score ≥ 3 as a stand-alone dose-reduction criterion. The 2010 ESC guidelines suggested that dabigatran 110 mg twice daily could be considered in patients at increased bleeding risk (e.g., HAS-BLED ≥ 3) [10]. Subsequently, the 2012 ESC focused update explicitly included a HAS-BLED score ≥ 3 among the considerations for lower doses of both dabigatran and rivaroxaban [11]. These historical recommendations formed the basis of the predefined anticoagulation eligibility criteria applied in our study. Moreover, renal function was assessed using MDRD-derived eGFR rather than creatinine clearance calculated using the Cockcroft–Gault equation, which is generally used for renal dose adjustment in product labelling.

The oral anticoagulation eligibility criteria remained unchanged throughout the recruitment period. Before discharge from the index hospitalization, anticoagulation therapy was reassessed and, whenever necessary, adjusted according to contemporary guideline recommendations.

### 2.3. Echocardiographic Assessment

All transthoracic and transesophageal echocardiographic examinations were performed by experienced echocardiographers using commercially available ultrasound systems (Vivid 7, Vivid E9, and Vivid E95; GE Healthcare, Horten, Norway). The examinations followed standardized institutional protocols and the contemporary echocardiographic recommendations applicable during the study period [15].

Comprehensive transthoracic echocardiography was performed before TEE to assess cardiac chamber dimensions, left ventricular systolic function, and valvular abnormalities. LVEF was calculated using the biplane Simpson method [16].

TEE was subsequently performed before the planned rhythm-control intervention to assess for LAAT. The left atrium and left atrial appendage were systematically examined in multiple imaging planes. LAAT was diagnosed when a well-defined echogenic mass, clearly distinguishable from the atrial endocardium and pectinate muscles, was visualized in at least two different imaging planes [17,18].

All echocardiographic variables, including LVEF, left atrial diameter, and the presence or absence of LAAT, were prospectively recorded during the index hospitalization and were subsequently used for statistical analyses.

### 2.4. Study Endpoints and Follow-Up

The primary endpoint was the presence of LAAT detected during the index TEE examination. Long-term clinical outcomes included cardiovascular death, all-cause death, hospitalization for HF, ischemic stroke, transient ischemic attack (TIA), myocardial infarction (MI), and systemic thromboembolism (STE). Cardiovascular death and HF hospitalization were the principal time-to-event outcomes and were further evaluated using Kaplan–Meier survival analysis and Cox proportional hazards regression models.

The study therefore distinguished the index TEE-based primary endpoint (LAAT) from the longitudinal clinical outcomes. Cardiovascular death and HF hospitalization were the principal long-term outcomes of interest, whereas the other clinical events were assessed as additional long-term outcomes. The observational analyses of long-term outcomes were intended to complement, rather than replace, the primary cross-sectional comparison of LAAT between AF and AFL. Both the index LAAT assessment and the long-term clinical endpoints were planned before the corresponding analyses were undertaken. The distinction between the index TEE endpoint and the longitudinal outcomes reflects the planned study objectives.

Participants were prospectively followed from the index date until the occurrence of the respective study endpoint or the last available follow-up. For each time-to-event analysis, participants who did not experience the respective endpoint were right-censored at the date of their last available follow-up. Participants who experienced the endpoint were assigned an event status of 1, with follow-up time calculated from the index date to the date of the event, whereas those without the endpoint were assigned an event status of 0 and remained in the risk set until censoring. Right censoring was incorporated in both Kaplan–Meier analyses and Cox regression models.

For HF hospitalization, time-to-event analyses were based on the first qualifying hospitalization after the index TEE; subsequent hospitalizations in the same patient were not included in these analyses.

Ischemic stroke, MI, TIA, STE, and hospitalization for HF were identified on the basis of hospital discharge diagnoses recorded in inpatient medical documentation. Hospitalization for HF was defined as an unplanned hospital admission due to signs and/or symptoms of new-onset HF or worsening of previously diagnosed HF.

Deaths were classified as cardiovascular or non-cardiovascular according to the available medical documentation. For in-hospital deaths, the classification was based on the principal diagnosis recorded during the terminal hospitalization. For deaths occurring outside the hospital, cardiovascular death was assigned in cases of unexplained sudden death or death preceded by symptoms suggestive of an acute cardiovascular event after the exclusion of fatal trauma, advanced malignancy, or severe acute infectious disease. Cause-of-death classification was performed by at least two study investigators through a joint review of the available medical documentation. The investigators were aware of the baseline AF/AFL classification; no formal independent or blinded endpoint adjudication was performed.

Clinical outcomes were identified using multiple complementary sources, including hospital medical records, outpatient documentation, national healthcare databases providing nationwide hospitalization coverage, and direct contact with patients or their relatives. Clinical outcomes were assessed by the study investigators using the available documentation, primarily hospital discharge diagnoses.

For nonfatal clinical events, ascertainment was based on the documented hospital discharge diagnosis rather than on a separate, centrally adjudicated assessment using uniform study-specific diagnostic criteria. The available documentation did not permit systematic verification of each diagnosis against contemporary event-specific definitions. For deaths outside hospital, an unexplained sudden death could be classified as cardiovascular in the absence of a documented alternative cause; this operational classification does not establish a confirmed cardiovascular mechanism. The same ascertainment procedures were applied to the AF and AFL groups, but assessors were not blinded to index arrhythmia type.

The date of each endpoint was recorded and used for time-to-event analyses.

### 2.5. Statistical Analysis

Categorical variables were summarized as frequencies and percentages. For quantitative baseline variables, distributional characteristics were assessed separately in the AF and AFL groups to guide the choice of descriptive summaries and between-group statistical tests. Approximately symmetric continuous variables were summarized as mean (standard deviation [SD]); skewed variables and the CHA_2_DS_2_-VA score were summarized as median (interquartile range [IQR]). Between-group comparisons of continuous variables used the two-sided independent-samples Student’s *t* test when comparison of means under approximately similar variances was appropriate, or Welch’s *t* test when unequal variances or distributional features warranted a more robust mean comparison. The CHA_2_DS_2_-VA score was compared using the two-sided Mann–Whitney U test. For skewed continuous variables analyzed using Welch’s test, means were compared between groups while medians (IQRs) were displayed as descriptive summaries; the two statistics address different aspects of the distributions. Categorical variables were compared using Pearson’s χ^2^ or Fisher’s exact test, as appropriate. All tests were two-sided, with *p* < 0.05 considered statistically significant. Absolute standardized mean differences (SMDs) were used to describe baseline imbalance: for continuous variables, SMDs were calculated from group means and SDs using the pooled variance denominator; for binary variables, differences in proportions were standardized using the corresponding average Bernoulli variances. SMDs were computed independently of the summary format displayed in Table 2.

No prespecified minimum clinically meaningful difference between AF and AFL was defined for LAAT or long-term clinical outcomes, and no formal sample-size calculation was performed to detect such a difference. The study was not designed to establish equivalence or non-inferiority between the arrhythmia groups.

The CHA_2_DS_2_-VA (HF, hypertension, age ≥ 75 years, diabetes mellitus, prior stroke/TIA/thromboembolism, vascular disease, and age 65–74 years) score was retrospectively recalculated for all participants according to the 2024 ESC guidelines by omitting the sex category component from the conventional CHA_2_DS_2_-VASc score [1].

This retrospective score calculation was undertaken to provide a uniform measure of baseline thromboembolic risk for cohort description and statistical adjustment. It does not imply that CHA_2_DS_2_-VA was available or used to guide clinical anticoagulation decisions throughout the 2010–2024 recruitment period; contemporaneous decisions were made under the recommendations and risk-assessment approaches applicable at the time.

The association between index arrhythmia type (AF versus AFL) and the primary endpoint of LAAT was evaluated using binary logistic regression. Univariable analyses were performed for LVEF, left atrial diameter, eGFR, CHA_2_DS_2_-VA score, and index arrhythmia type. A multivariable logistic regression model included LVEF, left atrial diameter, eGFR, CHA_2_DS_2_-VA score, and index arrhythmia type; AFL versus AF was forced into the model irrespective of its statistical significance in univariable analysis because it represented the primary exposure of interest. Results are reported as odds ratios (ORs) or adjusted odds ratios (aORs) with 95% confidence intervals (CIs). All 656 patients had complete data for variables included in the model.

Time-to-event analyses were performed for cardiovascular death and hospitalization for HF. The date of the index TEE examination was defined as time zero for all survival analyses. The median duration of follow-up was estimated using the reverse Kaplan–Meier method. Event-free survival was estimated using the Kaplan–Meier method, and survival distributions between patients with AF and AFL were compared using the log-rank test.

Associations between baseline characteristics and the risk of cardiovascular death and hospitalization for HF were initially assessed using univariable Cox proportional hazards regression models and expressed as hazard ratios (HRs) with 95% CIs. Multivariable Cox regression models were subsequently constructed to assess whether index arrhythmia type (AFL versus AF) was independently associated with each outcome after adjustment for clinically relevant covariates. Index arrhythmia type was retained in all multivariable models irrespective of its statistical significance. Multivariable analyses were performed using complete-case data; all 656 patients had complete data for the variables included in the final models. Detailed univariable results are reported in Supplementary Table S2.

Covariate inclusion in the final multivariable Cox models was guided by clinical relevance, established prognostic associations, and the potential for confounding, rather than by univariable *p*-value thresholds or automated stepwise selection. Index arrhythmia type (AFL versus AF) was the primary exposure of interest and was retained regardless of statistical significance. The endpoint-specific covariate sets reflected clinically relevant prognostic considerations; therefore, not every candidate variable was entered into both models. The final cardiovascular-death model contained 10 baseline covariate coefficients and one additional time-varying coefficient (11 fitted coefficients for 96 events), whereas the final HF-hospitalization model contained 14 baseline covariate coefficients and three additional time-varying coefficients (17 fitted coefficients for 91 events).

The CHA_2_DS_2_-VA score was included as a composite measure of baseline thromboembolic risk, whereas selected individual clinical covariates were retained to account for their potentially distinct prognostic associations with the study outcomes. Given the potential overlap between these variables, sensitivity analyses excluding the composite score were performed.

Multicollinearity among baseline covariates in the two primary survival models was assessed using variance inflation factors (VIFs), calculated from the baseline covariate design matrices. VIFs were recalculated after excluding CHA_2_DS_2_-VA to evaluate redundancy between the composite score and individual clinical covariates. This diagnostic did not include covariate-by-log(time) interaction terms and therefore does not constitute a complete assessment of collinearity in the extended Cox models (Supplementary Table S9).

Covariate selection for the LAAT logistic model and the cardiovascular death and HF hospitalization Cox models was informed by clinical relevance and potential baseline confounding, rather than by univariable *p* values alone. The LAAT model included index arrhythmia type, LVEF, left atrial diameter, eGFR, and CHA_2_DS_2_-VA score. The survival models additionally considered baseline cardiovascular history and clinical characteristics relevant to prognosis, as specified in Table 4; the alternative HF model and its covariates are detailed in Supplementary Table S1. Age and cardiovascular comorbidities were represented by the CHA_2_DS_2_-VA score and, where included, individual covariates; LVEF and left atrial diameter captured baseline cardiac function and remodeling. These adjustments address measured baseline differences but cannot eliminate residual confounding. Subsequent rhythm-control interventions were not modeled as baseline confounders, because treatment selection followed the index TEE and may have been influenced by arrhythmia type and TEE findings; their inclusion as fixed baseline exposures could introduce bias. The alternative model specifications in Supplementary Table S1 assess sensitivity to covariate selection, whereas the univariable associations in Supplementary Table S2 do not constitute confounder adjustment. The fitted survival models used the time-to-event and event-indicator variables in the form Surv(time, status), with follow-up measured in days from the index TEE.

The proportional hazards assumption was assessed using Schoenfeld residuals. When evidence of non-proportional hazards was identified, extended Cox regression models incorporating time-varying coefficients for the affected covariates were fitted, retaining the same adjustment covariates as in the corresponding conventional models. Time-dependent effects were modeled using tt(X,t) = X × log(t), where t denotes uncentered follow-up time in days and log denotes the natural logarithm, without an added constant. For these covariates, HR(t) = exp[β + γ log(t)], where β is the main coefficient and γ is the time-interaction coefficient. Consequently, exp(β) represents the hazard ratio at t = 1 day, not a constant effect throughout follow-up; γ quantifies the change in the log-hazard ratio per unit increase in log(t). A time-varying coefficient was included for history of HF in the cardiovascular-death model and for history of HF, LVEF, and previous stroke/TIA/STE in the HF-hospitalization model. Schoenfeld residual diagnostics and time-interaction estimates are reported in Supplementary Table S4.

For clinical interpretation, hazard ratios at 1, 3, 5, and 8 years were calculated for covariates with time-varying coefficients. The 95% confidence intervals were obtained from the variance of this linear combination, including the covariance between β and γ (Supplementary Table S7). These time-specific estimates describe modeled associations rather than causal effects.

The conventional and extended Cox models were compared using likelihood-ratio tests (LRTs) and the Akaike information criterion (AIC). Because covariates showing evidence of non-proportional hazards were explicitly modeled using time-varying coefficients, the proportional hazards assumption was not reassessed in the extended models.

As sensitivity analyses, the multivariable Cox models for cardiovascular death and HF hospitalization were refitted after exclusion of the CHA_2_DS_2_-VA score to assess the robustness of the association between index arrhythmia type and clinical outcomes across alternative model specifications. For HF hospitalization, an additional parsimonious multivariable Cox model using a reduced set of covariates was also fitted, with index arrhythmia type retained irrespective of statistical significance; non-proportional effects identified in this model were handled using the same time-varying coefficient approach described above.

As an exploratory index-management sensitivity analysis, the original extended Cox models for cardiovascular death and first HF hospitalization were refitted after excluding patients who underwent catheter ablation during the index hospitalization. The same baseline covariates and time-varying coefficient specifications as in the primary models were retained, with follow-up measured from index TEE. This restriction was not intended to estimate a treatment-independent causal effect, because index-hospitalization ablation was a post-enrollment management decision and later ablation exposure remained unaccounted for.

To evaluate potential confounding by secular changes in clinical management, we performed an additional sensitivity analysis incorporating the calendar year of the index TEE as a continuous covariate in the primary multivariable logistic regression model for LAAT and in both final extended Cox models for cardiovascular death and first HF hospitalization. All other model covariates and the time-varying coefficients were retained unchanged. The index TEE date defined the enrollment year. Results are presented in Supplementary Table S5.

To address death as a competing event for first HF hospitalization, we performed an exploratory competing-risk sensitivity analysis. The estimand was the subdistribution hazard of first HF hospitalization before death from any cause, comparing AFL with AF, with follow-up beginning at index TEE. Death before first HF hospitalization was treated as a competing event, whereas patients alive without HF hospitalization were censored at their last available follow-up. We fitted a Fine–Gray subdistribution hazards model with the same 14 baseline covariates as the primary HF model, but without time-varying coefficients, and a conventional cause-specific Cox model using these same baseline covariates for comparison. The Fine–Gray model used inverse-probability-of-censoring weights estimated from the overall censoring distribution; model-based 95% confidence intervals were calculated from the fitted information matrix. When HF hospitalization and death occurred on the same recorded day, HF hospitalization was classified as the first event in the primary sensitivity analysis, with the opposite convention assessed separately (Supplementary Table S8).

For cardiovascular death, we additionally performed a competing-risk sensitivity analysis treating non-cardiovascular death as the competing event. The estimand was the adjusted subdistribution hazard of cardiovascular death in the presence of non-cardiovascular mortality. A Fine–Gray model and a corresponding cause-specific Cox model were fitted with the same 10 baseline covariates as the primary cardiovascular-death model, without its time-varying coefficient. Patients alive at the end of their observed follow-up were censored (Supplementary Table S8).

Statistical analyses were performed using R (version 4.5.2; R Foundation for Statistical Computing, Vienna, Austria) and TIBCO Statistica, version 13.3 (TIBCO Software Inc., Palo Alto, CA, USA).

## 3. Results

### 3.1. Study Population

Between December 2010 and April 2024, 656 consecutive anticoagulated patients referred for a TEE-guided rhythm-control strategy were prospectively enrolled. The study population comprised 558 patients (85.1%) with AF and 98 patients (14.9%) with AFL.

The date of the index TEE examination defined the baseline (index date) and the start of follow-up for all time-to-event analyses.

During follow-up, cardiovascular death occurred in 96 patients (14.6%), hospitalization for HF in 91 (13.9%), ischemic stroke in 21 (3.2%), TIA in 7 (1.1%), MI in 16 (2.4%), and STE in 3 patients (0.5%). Overall, 111 patients (16.9%) died during the observation period. All 111 deaths were classified as cardiovascular (*n* = 96) or non-cardiovascular (*n* = 15); no deaths remained unclassified.

### 3.2. Baseline Characteristics of the Study Population

The overall cohort had a mean age of 65.4 (10.0) years, and 60.1% of participants were male. The mean body mass index was 30.0 (4.7) kg/m^2^, and the median CHA_2_DS_2_-VA score was 3. Hypertension was present in 79.0% of patients, a history of HF in 56.6%, diabetes mellitus in 25.2%, previous MI in 7.9%, and previous stroke, TIA, or STE in 7.6%.

The mean LVEF was 53.2 (11.8)%, and the mean left atrial diameter was 44.7 (5.1) mm. Reduced LVEF (<50%) was observed in 25.5% of patients, including 11.9% with severely reduced LVEF (LVEF < 40%). Left atrial enlargement (>40 mm) was present in 80.9% of the cohort, and TEE detected LAAT in 89 patients (13.6%), all of whom met the predefined anticoagulation eligibility criteria.

DOACs were the predominant anticoagulation strategy both on admission and at discharge, whereas VKAs were prescribed in approximately 11% of patients. Electrical cardioversion was performed during the index hospitalization in 503 patients (76.7%), whereas catheter ablation was performed during that hospitalization in 46 patients (7.0%), exclusively in the AFL group.

At hospital admission, 34 patients (5.2% of the overall cohort) were receiving dabigatran 110 mg twice daily and 21 patients (3.2%) were receiving rivaroxaban 15 mg once daily. These reduced-dose regimens were recorded in 41 patients with AF (7.3% of the AF group) and 14 patients with AFL (14.3% of the AFL group). A HAS-BLED score ≥ 3 was the recorded reason for dose reduction in two dabigatran-treated patients and four rivaroxaban-treated patients. LAAT was detected in 14 of the 55 patients receiving one of these regimens at admission. At the index TEE, LAAT was detected in 75 of 582 patients receiving DOACs (12.9%) and in 14 of 74 patients receiving VKAs (18.9%). The descriptive distribution of LAAT by individual anticoagulant and index arrhythmia type is provided in Supplementary Table S3. These unadjusted frequencies should not be interpreted as comparative estimates of anticoagulant effectiveness.

Detailed baseline demographic, clinical, laboratory, echocardiographic, and treatment characteristics are presented in Table 2.

### 3.3. Comparison of Baseline Characteristics Between AF and AFL

Patients with AFL were older than those with AF (mean 68.4 [SD 10.9] vs. 64.9 [SD 9.8] years, *p* = 0.001) and had a higher CHA_2_DS_2_-VA score (median 3 [IQR 2–4] vs. 3 [2,3], *p* = 0.001). At presentation, patients with AFL also had a higher heart rate (median 114.5 [IQR 84.2–130.8] vs. 90 [IQR 80–110] beats/min, Welch’s test *p* < 0.001). The proportion of patients aged ≥75 years was approximately twice as high in the AFL group as in the AF group (29.6% vs. 14.7%, *p* < 0.001).

No statistically significant between-group differences were observed in sex distribution, body mass index (BMI), systolic or diastolic blood pressure, left atrial diameter, LVEF, or eGFR. Mean eGFR was 64.1 (SD 15.8) in AFL and 67.3 (SD 15.3) mL/min/1.73 m^2^ in AF (Student’s *t* test, *p* = 0.06). Left atrial enlargement, defined as a left atrial diameter >40 mm, was more frequent in patients with AF than in those with AFL (82.6% vs. 71.4%, *p* = 0.009).

Among comorbidities, a history of HF was more frequent in patients with AF than in those with AFL (58.4% vs. 45.9%, *p* = 0.02), whereas diabetes mellitus was more frequent in patients with AFL (35.7% vs. 23.3%, *p* = 0.009). No significant differences were observed in the prevalence of hypertension, chronic obstructive pulmonary disease, previous MI, peripheral artery disease, aortic atherosclerosis, or previous stroke, TIA, or STE.

The distribution of oral anticoagulant therapy at admission and discharge, as well as the use of ACE inhibitors/angiotensin receptor blockers, beta-blockers, and statins at discharge, did not differ significantly between the AF and AFL groups. Group-specific characteristics are shown in Table 2.

### 3.4. Index Hospitalization and Rhythm-Control Strategy

LAAT was detected on index TEE in 89 patients (13.6%), resulting in postponement of the planned rhythm-control intervention. In these patients, oral anticoagulant therapy was either continued or switched to another oral anticoagulant, according to the treating physician’s decision and current guideline recommendations.

Electrical cardioversion was performed during the index hospitalization in 503 patients (76.7%). Catheter ablation during the index hospitalization was performed in 46 patients (7.0%), exclusively in the AFL group; none of these patients underwent electrical cardioversion during the index hospitalization. Among the remaining 107 patients who underwent neither electrical cardioversion nor index catheter ablation, 89 had LAAT detected on index TEE, whereas in the other 18 patients, a rhythm-control intervention was not performed because of the recurrent nature of the arrhythmia, at the discretion of the treating physician.

Overall, 219 patients (33.4%) underwent catheter ablation during the study period, including those treated during the index hospitalization.

### 3.5. Comparison of Primary and Secondary Study Endpoints Between AF and AFL

The comparison of study endpoints according to index arrhythmia type is presented in Table 2. The prevalence of LAAT detected at the index TEE did not differ significantly between patients with AF and AFL (14.2% vs. 10.2%, respectively; *p* = 0.29).

In univariable logistic regression, AFL versus AF was not associated with LAAT (OR 0.69, 95% CI 0.34–1.38; *p* = 0.29). In the multivariable model, with index arrhythmia type forced into the model, AFL remained not independently associated with LAAT (aOR 0.53, 95% CI 0.25–1.11; *p* = 0.09). Lower LVEF (aOR 0.95 per 1% increase, 95% CI 0.93–0.97; *p* < 0.001) and a higher CHA_2_DS_2_-VA score (aOR 1.41 per 1-point increase, 95% CI 1.18–1.69; *p* < 0.001) were independently associated with LAAT, whereas left atrial diameter and eGFR were not statistically significant after multivariable adjustment (Table 3).

The median follow-up, estimated using the reverse Kaplan–Meier method, was 2501 (1331–3116) days, corresponding to 6.85 (3.64–8.53) years.

The unadjusted between-group comparisons were not statistically significant for hospitalization for HF (14.0% vs. 13.3%, *p* = 0.85), cardiovascular death (14.0% vs. 18.4%, *p* = 0.26), MI (2.5% vs. 2.0%, *p* = 0.99), stroke (3.0% vs. 4.1%, *p* = 0.54), TIA (1.1% vs. 1.0%, *p* = 0.99), or STE (0.5% vs. 0%, *p* = 0.99).

The event proportions presented in Table 2 represent crude cumulative event frequencies within each arrhythmia group, whereas the Kaplan–Meier analyses and Cox regression models account for individual follow-up times and right censoring.

Comparisons of MI, ischemic stroke, TIA, and STE were descriptive and exploratory because of the small numbers of events; non-significant *p* values should not be interpreted as evidence of equivalent risks between AF and AFL.

All-cause mortality was numerically higher among patients with AFL than among those with AF (23.5% vs. 15.8%), although the difference did not reach statistical significance (*p* = 0.06).

Cardiovascular death and hospitalization for HF were the principal time-to-event outcomes and were further evaluated using Kaplan–Meier analysis and Cox proportional hazards regression. The corresponding multivariable Cox regression results are presented in Table 4.

### 3.6. Cardiovascular Death

**Figure 1:**
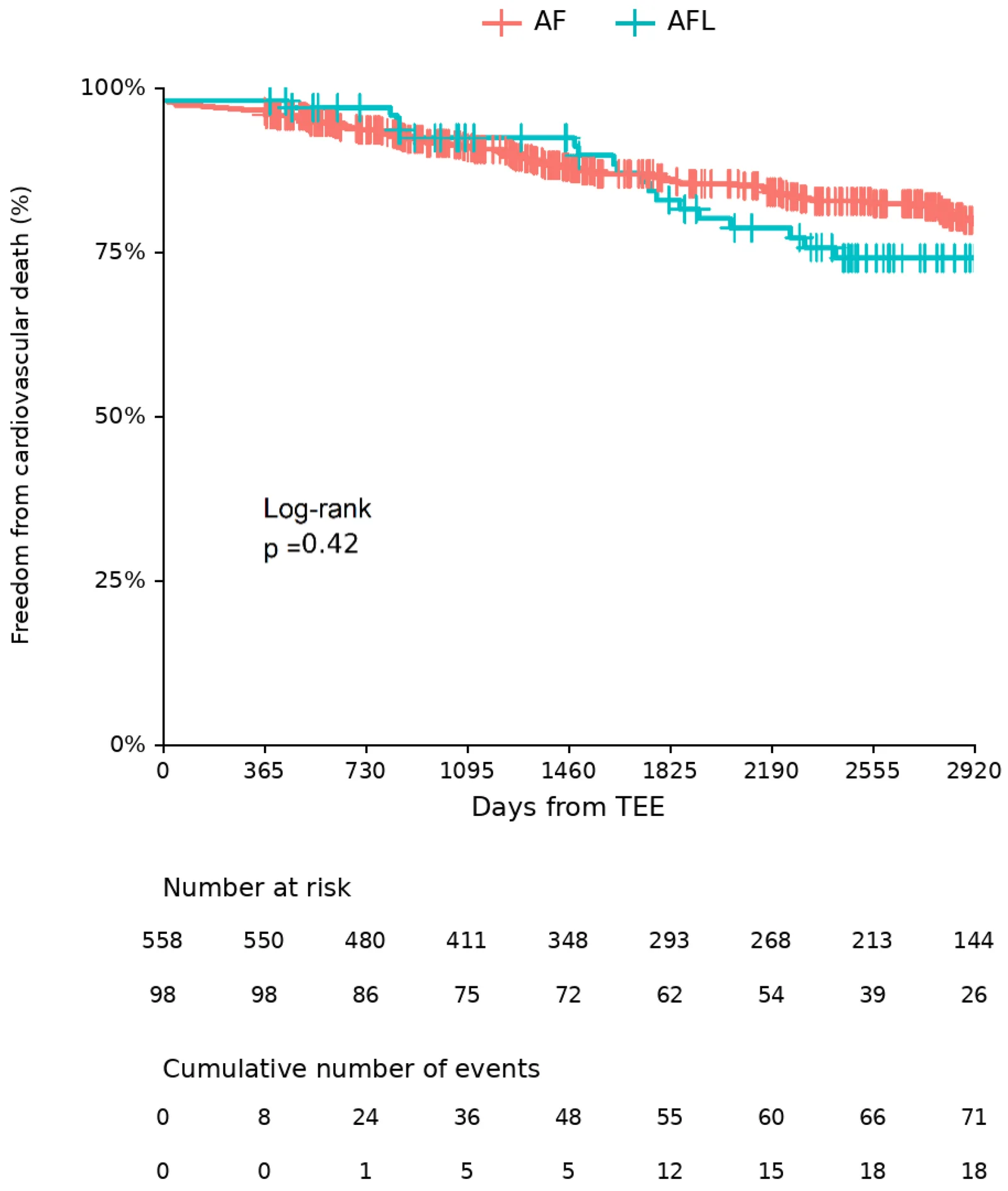
Kaplan–Meier curves for cardiovascular death according to index arrhythmia type. The log-rank comparison between patients with AF and AFL was not statistically significant (log-rank *p* = 0.42). Numbers at risk and cumulative numbers of events are shown below the graph. For graphical clarity, the Kaplan–Meier curves are displayed through 8 years (2920 days), whereas all time-to-event analyses were performed using the complete available follow-up. At 1, 3, 5, and 8 years, the numbers at risk were 550, 411, 293, and 144 for AF and 98, 75, 62, and 26 for AFL, respectively. Abbreviations: AF, atrial fibrillation; AFL, atrial flutter; and TEE, transesophageal echocardiography.

The log-rank comparison of Kaplan–Meier curves for cardiovascular death between patients with AF and AFL was not statistically significant (log-rank *p* = 0.42; Figure 1). During follow-up, cardiovascular death occurred in 96 patients. In univariable Cox regression analysis, index arrhythmia type was not associated with cardiovascular mortality (AFL vs. AF: HR 1.23, 95% CI 0.74–2.06; *p* = 0.42).

In the initial multivariable Cox model, AFL was not independently associated with cardiovascular death compared with AF (HR 1.07, 95% CI 0.63–1.83; *p* = 0.79). Testing of the proportional hazards assumption showed no evidence of non-proportionality for index arrhythmia type (*p* = 0.21) or for the model globally (*p* = 0.19); however, the proportional hazards assumption was violated for a history of HF (*p* = 0.04). Of the 96 cardiovascular deaths, 78 occurred in patients with AF and 18 in patients with AFL. Numbers at risk at 1, 3, 5, and 8 years are provided in the Figure 1 legend; estimates at later time points should be interpreted cautiously because relatively few patients with AFL remained at risk.

An extended Cox model incorporating a time-varying effect for history of HF was therefore fitted. In this model, index arrhythmia type was not independently associated with cardiovascular death (AFL vs. AF: adjusted hazard ratio [aHR] 1.09, 95% CI 0.64–1.85; *p* = 0.75). LAAT (aHR 1.71, 95% CI 1.04–2.81; *p* = 0.03) and lower LVEF (aHR 0.97 per 1% increase, 95% CI 0.96–0.99; *p* = 0.003) were associated with cardiovascular death. Peripheral artery disease also showed an association (aHR 4.56, 95% CI 1.51–13.81; *p* = 0.007), although this estimate should be considered exploratory because only nine participants had this condition.

The time-varying component for history of HF was significant (HR for the time interaction 0.452, 95% CI 0.220–0.929; *p* = 0.03), consistent with a finding that the prognostic association of baseline heart failure with cardiovascular mortality attenuated as follow-up progressed. Incorporation of this time-varying effect significantly improved model fit compared with the conventional Cox model (LRT *p* = 0.01; AIC 1089.66 vs. 1093.90). Detailed multivariable Cox regression results are presented in Table 4, whereas univariable results are provided in Supplementary Table S2.

The corresponding adjusted HRs for baseline history of HF were 3.208 (95% CI 1.089–9.450), 1.342 (0.780–2.308), 0.895 (0.511–1.568), and 0.616 (0.289–1.313) at 1, 3, 5, and 8 years, respectively (Supplementary Table S7). These estimates are imprecise, and the apparent decline should not be interpreted as evidence of a protective effect at later time points.

In the baseline-covariate VIF assessment, the maximum VIF for the cardiovascular-death model was 2.32 (CHA_2_DS_2_-VA), decreasing to 1.38 after removal of the score (Supplementary Table S9).

In a sensitivity analysis excluding the CHA_2_DS_2_-VA score from the multivariable model, index arrhythmia type remained not independently associated with cardiovascular death (AFL vs. AF: aHR 1.15, 95% CI 0.68–1.96; *p* = 0.61), with no material change in the estimate after exclusion of the CHA_2_DS_2_-VA score. These results are summarized in Supplementary Table S1.

### 3.7. Heart Failure Hospitalization

**Figure 2:**
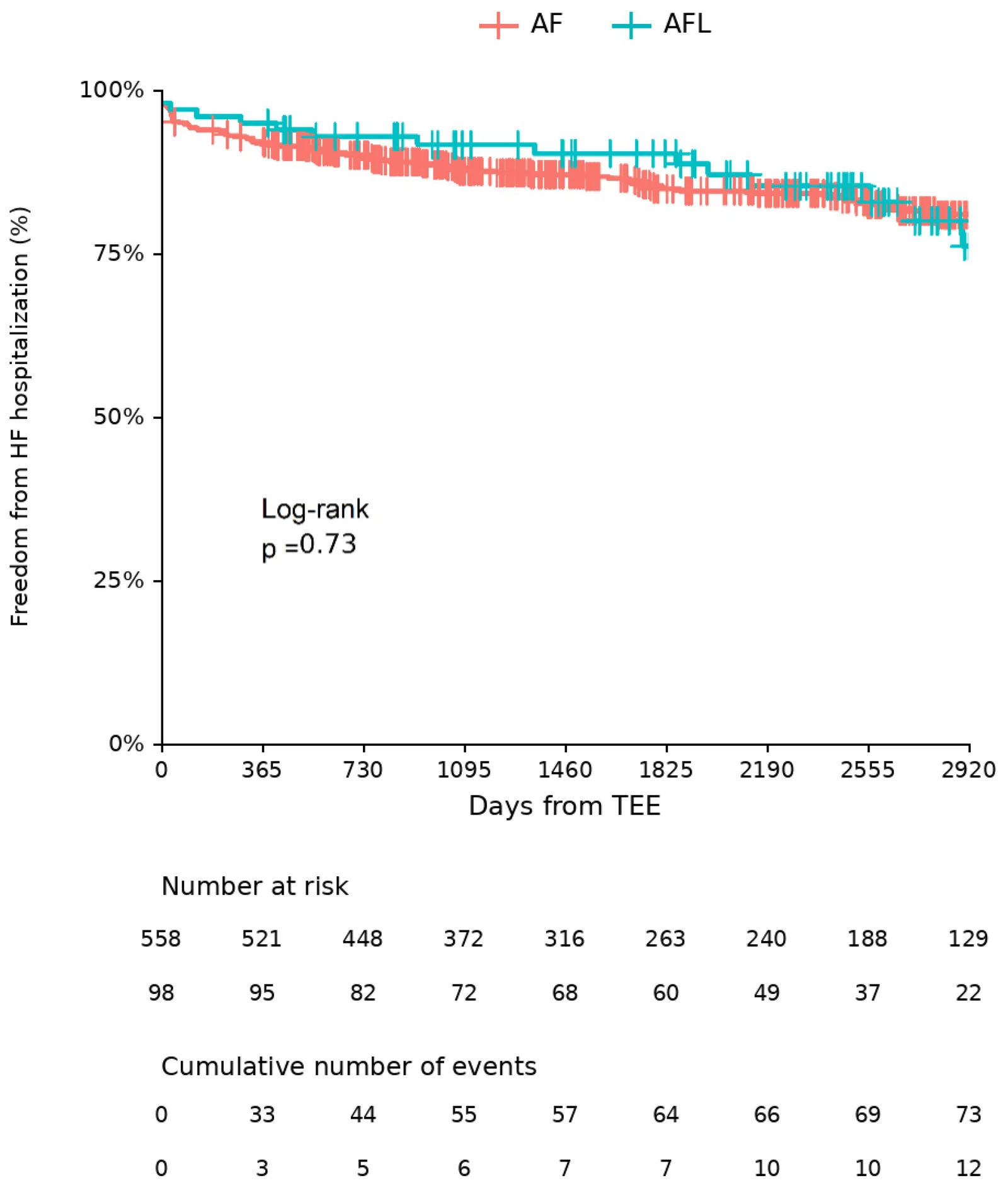
Kaplan–Meier curves for HF hospitalization according to index arrhythmia type. The log-rank comparison between patients with AF and AFL was not statistically significant (log-rank *p* = 0.73). Numbers at risk and cumulative numbers of events are shown below the graph. For graphical clarity, the Kaplan–Meier curves are displayed through 8 years (2920 days), whereas all time-to-event analyses were performed using the complete available follow-up. At 1, 3, 5, and 8 years, the numbers at risk were 521, 372, 263, and 129 for AF and 95, 72, 60, and 22 for AFL, respectively. Abbreviations: AF, atrial fibrillation; AFL, atrial flutter; HF, heart failure; TEE, transesophageal echocardiography.

Kaplan–Meier analysis showed that the log-rank comparison for HF hospitalization between patients with AF and AFL was not statistically significant (log-rank *p* = 0.73; Figure 2).

During follow-up, 91 patients were hospitalized for HF (78 with AF and 13 with AFL). Numbers at risk at 1, 3, 5, and 8 years are provided in the Figure 2 legend; late estimates, particularly in the AFL subgroup, are less precise. In univariable Cox regression analysis, index arrhythmia type was not associated with the risk of HF hospitalization (AFL vs. AF: HR 0.90, 95% CI 0.50–1.62; *p* = 0.73). Full univariable Cox regression results are provided in Supplementary Table S2.

In the initial multivariable Cox model, AFL was not independently associated with HF hospitalization compared with AF (HR 0.68, 95% CI 0.37–1.27; *p* = 0.23). The proportional hazards assumption was satisfied for index arrhythmia type (*p* = 0.16) but was violated for LVEF (*p* = 0.002), previous stroke/TIA/STE (*p* = 0.01), and a history of HF (*p* = 0.006). The global test also indicated violation of the proportional hazards assumption (*p* = 0.009).

An extended Cox model incorporating time-varying effects for LVEF, previous stroke/TIA/STE, and history of HF was therefore fitted. After accounting for these time-dependent effects, index arrhythmia type was not independently associated with HF hospitalization (AFL vs. AF: aHR 0.69, 95% CI 0.37–1.27; *p* = 0.23). Among covariates with proportional effects, lower eGFR (aHR 0.97 per 1 mL/min/1.73 m^2^ increase, 95% CI 0.96–0.99; *p* = 0.002) and greater left atrial diameter (aHR 1.10 per 1 mm increase, 95% CI 1.06–1.15; *p* < 0.001) were associated with the outcome. Peripheral artery disease was also associated with HF hospitalization (aHR 5.81, 95% CI 1.60–21.06; *p* = 0.008), but this estimate should be interpreted as exploratory given the small number of participants with this condition.

The extended model provided a significantly better fit than the conventional Cox model (LRT *p* = 0.002), with a reduction in AIC from 1011.29 to 1002.25. The time-varying component for previous stroke/TIA/STE was significant (HR for the time interaction 1.825, 95% CI 1.038–3.208; *p* = 0.04), consistent with an increase over time in its prognostic association with subsequent HF hospitalization. The individual time-varying components for history of HF and LVEF were not statistically significant after simultaneous modeling, although their inclusion formed part of the extended model selected on the basis of non-proportional hazards and improved overall fit (Table 4).

Time-specific adjusted HRs for previous stroke/TIA/STE were 1.117 (95% CI 0.493–2.532), 2.163 (1.128–4.148), 2.941 (1.381–6.263), and 3.901 (1.548–9.831) at 1, 3, 5, and 8 years, respectively (Supplementary Table S7). The LVEF × log(time) interaction was not statistically significant (*p* = 0.21); its inclusion was based on the proportional-hazards assessment and should not be taken as evidence of a confirmed time-dependent LVEF association.

In a further sensitivity analysis adjusting for the calendar year of index TEE, the association of AFL versus AF remained similar for cardiovascular death (aHR 1.09, 95% CI 0.64–1.85; *p* = 0.75) and first HF hospitalization (aHR 0.68, 95% CI 0.37–1.27; *p* = 0.23; Supplementary Table S5). The adjusted association with LAAT was likewise similar (aOR 0.54, 95% CI 0.26–1.14; *p* = 0.11).

In the baseline-covariate VIF assessment, the maximum VIF for the HF-hospitalization model was 5.39 (CHA_2_DS_2_-VA), decreasing to 1.49 after removal of the score (Supplementary Table S9). These diagnostics do not assess additional collinearity involving time-varying coefficient terms.

In a descriptive analysis by recruitment era (2010–2014, 2015–2019, and 2020–2024), the proportions of patients with AFL were 12.5% (7/56), 19.0% (66/348), and 9.9% (25/252), respectively. Catheter ablation at any time during the study was recorded in 16.3% (8/49), 18.8% (53/282), and 33.5% (76/227) of patients with AF, compared with 85.7% (6/7), 83.3% (55/66), and 84.0% (21/25) of patients with AFL, respectively. Among patients with AFL, index-hospitalization ablation occurred in 14.3% (1/7), 54.5% (36/66), and 36.0% (9/25), respectively; no patient with AF underwent index-hospitalization ablation. Because later ablation exposure depended in part on the duration of available follow-up, these descriptive proportions should not be interpreted as calendar-year-specific ablation rates.

The finding that index arrhythmia type was not independently associated with HF hospitalization was consistent across sensitivity analyses using alternative model specifications. In a more parsimonious multivariable model, AFL versus AF was not independently associated with HF hospitalization after accounting for time-varying effects of LVEF and history of HF (aHR 0.76, 95% CI 0.42–1.40; *p* = 0.38). Similarly, after exclusion of the CHA_2_DS_2_-VA score from the full multivariable model, the association remained materially unchanged (aHR 0.69, 95% CI 0.37–1.28; *p* = 0.24). These sensitivity analyses are summarized in Supplementary Table S1.

For cardiovascular death, 96 patients died from cardiovascular causes, 15 died from non-cardiovascular causes, and 545 were censored alive. In the 10-covariate cause-specific Cox sensitivity model, the adjusted AFL-versus-AF HR was 1.07 (95% CI 0.63–1.83; *p* = 0.79). The corresponding Fine–Gray subdistribution HR was 1.07 (95% CI 0.63–1.83; *p* = 0.79), compared with 1.09 (95% CI 0.64–1.85; *p* = 0.75) in the primary extended Cox model (Supplementary Table S8).

In the competing-risk analysis of first HF hospitalization, 91 patients experienced a qualifying HF hospitalization, 75 died before any HF hospitalization, and 490 were censored without either event. Two patients had HF hospitalization and death recorded on the same day. In the 14-covariate cause-specific Cox sensitivity model, the adjusted AFL-versus-AF HR was 0.68 (95% CI 0.37–1.27; *p* = 0.23). The corresponding Fine–Gray subdistribution HR was 0.70 (95% CI 0.38–1.30; *p* = 0.26), with no statistically significant association (Supplementary Table S8). Classifying the two same-day events as competing deaths instead yielded a subdistribution HR of 0.71 (95% CI 0.38–1.33; *p* = 0.29).

## 4. Discussion

In this cohort, index arrhythmia type (AF versus AFL) was not significantly associated with cardiovascular death or HF hospitalization after multivariable adjustment, including time-varying effects where required. It was also not significantly associated with LAAT at the index TEE. However, the precision of the AF-versus-AFL estimates was limited, and these findings should not be interpreted as evidence of equivalent risks.

Interpretation of these findings requires consideration of an important methodological issue that has limited previous comparisons between AF and AFL. Much of the available evidence has been derived either from nationwide administrative registries [4,19,20] or from smaller clinical studies [21], with substantial differences in patient selection, anticoagulation status, and baseline characterization. Consequently, the independent contribution of index arrhythmia type to long-term prognosis has remained uncertain. In contrast, the present study prospectively enrolled consecutive patients managed according to a predefined diagnostic pathway. All participants met predefined anticoagulation eligibility criteria, underwent systematic transesophageal echocardiography before planned rhythm-control intervention, and entered follow-up from the same clinically relevant time point. These features provide a consistent framework for evaluating the independent prognostic significance of index arrhythmia type while reducing some sources of heterogeneity in observational comparisons.

Two distinct analytical questions should be distinguished. The cross-sectional logistic regression analysis evaluated factors associated with LAAT detected at the index TEE, whereas the longitudinal Cox regression analyses evaluated baseline factors associated with subsequent cardiovascular death and HF hospitalization. Associations identified in the LAAT analysis should not be assumed to extend to long-term clinical outcomes, and vice versa.

### 4.1. AF Versus AFL and Long-Term Cardiovascular Outcomes

The relationship between index arrhythmia type and long-term prognosis remains controversial. Previous studies have reported inconsistent findings, ranging from significantly worse outcomes among patients with AF to a lack of statistically significant adjusted associations between arrhythmia subtype and prognosis after adjustment for baseline clinical characteristics [4,19,20,21]. More recent population-based data have further reported that both AF and AFL are associated with substantial long-term cardiovascular morbidity following hospitalization [6].

Large administrative database studies have generally reported less favorable outcomes among patients with AF than among those with AFL [4,19]. In a nationwide cohort study, Lin et al. demonstrated significantly higher risks of ischemic stroke, heart failure hospitalization, and all-cause mortality in patients with AF, with these differences persisting after propensity-score matching. Although these studies included large patient populations, patient classification relied on administrative diagnostic coding without standardized echocardiographic assessment, systematic evaluation for LAAT, or detailed information regarding anticoagulation quality and rhythm-control management. Consequently, residual differences in baseline clinical characteristics and patient management cannot be completely excluded as contributors to the observed prognostic differences [4,19].

The VIF findings indicated greater baseline-covariate redundancy in the HF-hospitalization model, primarily involving CHA_2_DS_2_-VA. The broadly similar AFL-versus-AF estimates after removing the score provide reassurance regarding this exposure estimate, but do not establish stability of every model coefficient or exclude collinearity involving time-dependent interactions (Supplementary Tables S1 and S9).

Other clinical studies have provided a different perspective. LeLorier et al. found no statistically significant adjusted differences in the long-term risks of stroke or mortality between patients with AF and AFL after adjustment for baseline clinical characteristics. During follow-up, 27.6% of patients initially presenting with AFL subsequently developed AF, suggesting that the presenting arrhythmia may not represent a stable long-term disease phenotype. Similarly, Chen et al. reported more favorable thromboembolic outcomes among patients with solitary AFL than among those with AF, whereas patients with AFL who subsequently developed AF had outcomes approaching those observed in AF. Together, these findings suggest that long-term prognosis may depend not only on the arrhythmia documented at presentation but also on subsequent arrhythmia evolution and the underlying cardiovascular disease process [20,21].

Our prospective cohort provides a standardized TEE-based evaluation of anticoagulated patients referred for rhythm control. No statistically significant adjusted association between index arrhythmia type and the principal long-term outcomes was observed, including in extended Cox models addressing non-proportional hazards.

The finding that index arrhythmia type was not independently associated with the principal long-term outcomes was consistent across alternative model specifications. Exclusion of the CHA_2_DS_2_-VA score did not materially alter the association for either cardiovascular death or HF hospitalization, and the HF finding was additionally observed in a more parsimonious multivariable model. These sensitivity analyses support the consistency of the primary finding across reasonable changes in covariate specification. The corresponding estimates are provided in Supplementary Table S1.

In the exploratory analysis excluding patients who underwent catheter ablation during the index hospitalization, 610 patients remained (558 with AF and 52 with AFL). Cardiovascular death occurred in 78 AF and 12 AFL patients, and first HF hospitalization occurred in 78 AF and 9 AFL patients. Adjusted AFL-versus-AF estimates were 1.33 (95% CI 0.72–2.49; *p* = 0.37) for cardiovascular death and 0.85 (95% CI 0.41–1.77; *p* = 0.67) for first HF hospitalization (Supplementary Table S6). Point estimates shifted relative to the full-cohort analysis, but confidence intervals remained wide and included unity.

The multivariable analyses suggested associations between several baseline cardiovascular characteristics and long-term outcomes. In the final model for cardiovascular death, LAAT and lower LVEF were associated with the outcome, whereas the prognostic association of baseline history of HF decreased over time. For HF hospitalization, lower eGFR and greater left atrial diameter showed significant proportional associations with risk. Although peripheral artery disease yielded statistically significant adjusted estimates for both outcomes, only nine participants had this condition (eight with AF and one with AFL). The wide confidence intervals indicate substantial imprecision; these findings should therefore be regarded as exploratory and hypothesis-generating, rather than robust evidence of an independent prognostic association. In addition, previous stroke/TIA/STE demonstrated a significant time-dependent association with HF hospitalization, with its prognostic association increasing during follow-up. History of HF and LVEF also required time-varying modeling for this outcome because the proportional hazards assumption was violated, although their individual time-varying components were not statistically significant in the full extended model. Overall, these observational findings suggest that selected baseline cardiovascular characteristics may contribute to risk assessment, but their associations vary in precision and should not be interpreted as establishing relative prognostic importance or causality.

From a clinical perspective, the time-varying coefficient of baseline history of HF in the cardiovascular death model suggests that its prognostic association was strongest earlier during follow-up and progressively attenuated over time. For HF hospitalization, the significant time-dependent association of previous stroke/TIA/STE suggests increasing prognostic relevance as follow-up progressed; conversely, although history of HF and LVEF required time-varying modeling because of non-proportional hazards, their individual time-varying components were not statistically significant in the full extended model, precluding a similarly specific directional interpretation. These temporal patterns should be interpreted as changes in prognostic associations over time rather than as evidence of causal changes in the effects of the underlying clinical conditions.

Time-specific HR estimates and their uncertainty are provided in Supplementary Table S7. The estimates for individual follow-up times are descriptive functions of the fitted model and should not be overinterpreted, especially at later time points. In particular, the LVEF × log(time) interaction for HF hospitalization was not statistically significant (*p* = 0.21), despite its retention following proportional-hazard diagnostics.

### 4.2. Left Atrial Appendage Thrombus

The analysis of LAAT at baseline was performed separately from the time-to-event analyses of long-term cardiovascular outcomes.

Although LAAT was numerically more frequent in patients with AF than in those with AFL (14.2% vs. 10.2%), this difference was not statistically significant. Index arrhythmia type was also not independently associated with LAAT after adjustment for LVEF, left atrial diameter, eGFR, and CHA_2_DS_2_-VA score (AFL vs. AF: aOR 0.53, 95% CI 0.25–1.11; *p* = 0.09). Lower LVEF and a higher CHA_2_DS_2_-VA score remained independently associated with LAAT. This finding suggests that, among patients meeting predefined anticoagulation eligibility criteria and undergoing systematic TEE before a planned rhythm-control intervention, index arrhythmia type alone may have limited value in identifying patients with LAAT.

Previous studies evaluating LAAT in patients with AFL have yielded heterogeneous results, with substantial differences in patient selection, anticoagulation status, imaging protocols, and the inclusion of patients with concomitant or previously documented AF. A systematic review by Vadmann et al. reported considerable variability in the reported prevalence of LAAT, spontaneous echo contrast, and thromboembolic events in patients with AFL, highlighting the difficulty of direct comparisons across studies with different clinical and methodological characteristics [22].

Early TEE-based studies demonstrated that atrial thrombi may occur in patients with AFL, but reported prevalence varied considerably across study populations. Bikkina et al. identified intra-atrial thrombus in 5 of 24 patients with AFL, whereas Irani et al. detected LAAT in 3 of 47 non-anticoagulated patients undergoing TEE before cardioversion. In a larger retrospective study, Parikh et al. analyzed 455 TEE examinations performed in 373 patients with AFL before ablation or cardioversion and identified left atrial thrombi in 5.3% of examinations. Anticoagulation strategies and eligibility criteria differed substantially among these studies, limiting direct comparison with a cohort meeting predefined anticoagulation eligibility criteria [23,24,25].

More recent direct comparisons have suggested a lower prevalence of LAAT in AFL than in AF. Huang et al. reported LAAT in 11% of patients with AF but in none of those with AFL and also observed more favorable indices of left atrial appendage mechanical function in AFL. However, patients receiving chronic oral anticoagulation were excluded from that study, limiting direct comparison with the present cohort [26].

In a systematic review and meta-analysis of 72 studies including 20,516 patients with non-valvular AF undergoing TEE, Di Minno et al. reported a weighted mean prevalence of left atrial thrombus of 9.8% (95% CI, 7.6–12.5%). Prevalence varied substantially across studies according to patient characteristics, AF type, and anticoagulation status, underscoring the heterogeneity of thrombus prevalence in AF populations [27].

Our previous prospective studies provide additional context for the present findings. Among 500 patients with AF receiving oral anticoagulation and referred for TEE-guided cardioversion, LAAT was detected in 13% of patients [28]. In a separate prospective cohort of patients with AFL receiving oral anticoagulation and managed with a similar TEE-guided rhythm-control strategy, LAAT was identified in 10% of patients [29]. The AF and AFL cohorts analyzed in the present study partially overlap with those included in our previous reports [28,29], while the current cohort also includes additional patients enrolled during the extended recruitment period. The present study extends these previous reports by integrating AF and AFL within a single analytical framework, using a common index time point and standardized eligibility and imaging criteria, and directly comparing LAAT and long-term clinical outcomes between the two arrhythmias.

The prognostic significance of LAAT in patients already receiving OAC remains less certain. In our previous AF cohort, LAAT was independently associated with cardiovascular mortality, whereas no statistically significant associations with stroke, TIA, or STE were observed [28]. In the AFL cohort, LAAT was not significantly associated with cardiovascular death or HF hospitalization, while the number of thromboembolic events was limited [29]. Thus, our previous studies did not demonstrate a significant association between LAAT and subsequent thromboembolic events during continued OAC therapy. However, the limited number of such events precludes concluding that LAAT has no prognostic relevance for thromboembolic outcomes.

These observations suggest that the clinical significance of LAAT in anticoagulated patients may be more complex than its conventional interpretation solely as a marker of thromboembolic risk. In our previous AF cohort, patients with LAAT had greater left atrial diameter, lower LVEF, and a higher prevalence of HF and previous MI [28]. Consistent with this interpretation, a subsequent analysis of the same AF cohort found no significant association between BMI and long-term cardiovascular outcomes, while highlighting the prognostic relevance of lower LVEF, impaired renal function, greater left atrial diameter, and LAAT reported in our earlier analysis [28,30]. Similarly, in the AFL cohort, patients with LAAT had greater left atrial diameter and more frequently had a history of stroke, TIA, or STE [29]. Taken together, these findings support the hypothesis that LAAT may coexist with a greater burden of structural and clinical cardiovascular abnormalities and may therefore also represent a marker of a more advanced atrial and cardiovascular disease phenotype.

Left atrial diameter alone may not fully characterize the underlying atrial disease substrate. Atrial cardiomyopathy can involve structural and functional abnormalities, including fibrosis, for which cardiac magnetic resonance may offer additional characterization beyond conventional echocardiographic measurements, although methodological limitations and incomplete standardization remain [31].

Neither the present study nor our previous cohorts included a comparison with patients not meeting the predefined oral anticoagulation criteria and therefore cannot determine the effect of OAC itself on the occurrence of LAAT. Furthermore, the limited number of thromboembolic events precludes robust conclusions regarding the independent association between LAAT and subsequent stroke or systemic embolism during continued OAC therapy. Accordingly, although our previous studies did not demonstrate a significant association between LAAT and subsequent thromboembolic events, its prognostic significance in patients meeting study-defined anticoagulation criteria, particularly with respect to thromboembolic outcomes, remains to be fully established. Consistent with this interpretation, LAAT remained independently associated with cardiovascular death in the present multivariable analysis, even after accounting for non-proportional effects of other covariates.

The association between LAAT and cardiovascular mortality should also be considered in the context of the clinical decisions prompted by its detection. In the present cohort, LAAT led to postponement of the planned rhythm-control intervention and continuation of anticoagulation, with a change in anticoagulant agent in some patients. Thus, LAAT may reflect the underlying cardiovascular risk profile while also influencing subsequent management. Given the observational design, its association with cardiovascular mortality should be interpreted cautiously and considered hypothesis-generating. This association should not be interpreted as evidence of a direct causal effect.

### 4.3. Clinical Interpretation of the Findings

AF and AFL differ in electrophysiological mechanisms and treatment approaches, but share important cardiovascular comorbidities. In this observational cohort, the adjusted analyses did not identify a statistically significant association between index arrhythmia type and long-term outcomes; the confidence intervals nevertheless allow for clinically meaningful differences.

AF and AFL may represent different clinical manifestations within a substantially overlapping atrial and cardiovascular disease substrate. Population-based studies have demonstrated that, despite differences in baseline clinical characteristics, both arrhythmias share many cardiovascular risk factors and are closely associated with HF and other cardiovascular comorbidities [7,9].

Structural atrial remodeling, ventricular dysfunction, hypertension, coronary artery disease, chronic kidney disease, diabetes mellitus, and HF may contribute both to the development of atrial arrhythmias and to subsequent adverse cardiovascular outcomes. Recent epidemiological data further indicate the substantial and increasing contribution of both AF and AFL to the global burden of HF [5].

The dynamic relationship between AF and AFL further supports this interpretation. Previous studies have shown that a substantial proportion of patients initially presenting with AFL subsequently develop AF and that the occurrence of AF during follow-up is associated with a clinical course increasingly resembling that observed in patients presenting with AF [20,21]. Accordingly, the arrhythmia documented at presentation may not represent a fixed long-term disease phenotype but rather one manifestation of an evolving atrial disease process.

Associations with subsequent outcomes were observed for several baseline cardiovascular characteristics, including LAAT and LVEF for cardiovascular death and eGFR and left atrial diameter for HF hospitalization; estimates based on rare covariates, particularly PAD, warrant additional caution. These associations may inform risk assessment alongside index arrhythmia type, but do not establish that the underlying cardiovascular phenotype is more prognostically important than AF-versus-AFL classification.

The infrequent secondary outcomes, particularly TIA and STE, were evaluated descriptively; the limited event counts preclude firm conclusions regarding between-group differences in their risks.

AF and AFL should not be regarded as clinically interchangeable: their electrophysiological mechanisms and rhythm-control strategies differ, particularly regarding cavotricuspid isthmus ablation for typical AFL [1,2]. Moreover, differences in treatment exposure and residual confounding limit causal interpretation of the observed outcomes.

Clinical assessment should therefore consider both the index arrhythmia type and the broader cardiovascular profile, while recognizing the uncertainty of the adjusted between-group estimates.

Beyond arrhythmia recurrence and major cardiovascular events, patient-centered outcomes—including symptoms, quality of life, treatment burden, and patient-reported experience—are important dimensions of rhythm-control care. These outcomes were not systematically assessed in our cohort and should be incorporated into future AF-versus-AFL comparisons [32].

Differences in rhythm-control pathways may also have implications for healthcare utilization and costs, including repeat procedures and hospital admissions. Although hospitalization outcomes were assessed in this study, resource use and economic endpoints were not systematically evaluated; these represent additional priorities for future comparative research [33].

### 4.4. Strengths and Limitations

The present study has several methodological strengths. Unlike many previous investigations comparing AF and AFL, it prospectively enrolled consecutive patients managed according to a predefined clinical pathway. All participants met predefined anticoagulation eligibility criteria and underwent systematic transesophageal echocardiography before a planned rhythm-control intervention, providing standardized baseline characterization and uniform assessment of LAAT. Furthermore, the date of the index TEE served as a common and clinically relevant starting point for follow-up in both groups. These features provided greater methodological consistency with respect to anticoagulation status, imaging strategy, and definition of the index date than in many previous observational comparisons. Finally, multivariable logistic regression allowed adjusted evaluation of the primary LAAT endpoint, while multivariable Cox regression allowed the association between index arrhythmia type and the principal long-term outcomes to be evaluated after accounting for clinically relevant differences in baseline cardiovascular risk. The proportional hazards assumption was formally assessed, and extended Cox models with time-varying coefficients were applied when required to account for non-proportional covariate effects.

Several limitations should also be acknowledged. First, this was a single-center observational study; therefore, residual and unmeasured confounding cannot be excluded despite prospective enrollment and multivariable adjustment. Although the cohort was clinically well characterized, the number of patients with AFL was substantially smaller than the number with AF, and the number of individual outcome events was limited for several endpoints. Consequently, robust multivariable analysis of the less frequent outcomes, including stroke, TIA, MI, and STE, was not feasible.

The study population was restricted to patients referred for TEE-guided rhythm-control evaluation who met the predefined anticoagulation eligibility criteria. Referral for rhythm-control management may have been influenced by symptom burden, comorbidities, and clinical decision-making. These selection processes may have introduced selection bias and may limit the generalizability of our findings beyond the enrolled population. Multivariable adjustment within the cohort cannot eliminate bias arising from patient selection.

The exclusion of patients with a history of previous cardioversion or catheter ablation for AF or AFL further restricted the study population. Consequently, the findings may not be generalizable to the broader AF and AFL populations, particularly patients who have previously undergone rhythm-control interventions.

Second, systematic rhythm surveillance was not performed during follow-up. Consequently, recurrent atrial arrhythmias and progression from AFL to AF may have remained undetected in some patients. This is particularly relevant because previous studies have reported that AF frequently develops during follow-up in patients initially presenting with AFL and may influence their subsequent clinical course [20,21]. As emphasized in the recent review by Grigoriou et al. [34], AF burden provides information beyond categorical rhythm classification and may be relevant to cardiovascular prognosis. Because AF burden was not systematically measured in our cohort, we could not examine its longitudinal changes or account for it in the outcome models.

Accordingly, the occurrence and timing of incident AF among patients presenting with AFL, the frequency of recurrent AF or AFL, and longitudinal atrial arrhythmia burden could not be reliably quantified. The index rhythm classification therefore should not be interpreted as a stable rhythm phenotype throughout follow-up. Because subsequent arrhythmia burden may be associated with cardiovascular outcomes and may differ according to rhythm-control treatment, its unmeasured contribution could not be evaluated or adjusted for in the prognostic models.

The interpretation of outcomes after rhythm-control interventions is also complicated by subsequent anticoagulation management. Evidence from a large systematic review and meta-analysis of oral anticoagulation after AF ablation illustrates the need to consider thromboembolic and major bleeding outcomes together. However, these findings cannot be directly extrapolated to our cohort of patients with AF and AFL, in which anticoagulation discontinuation and bleeding outcomes were not evaluated [35].

Electrical cardioversion and catheter ablation performed after the index TEE were not included as treatment covariates in the long-term outcome models. Such interventions may be consequences of index arrhythmia classification and clinical decision-making, and may in turn affect prognosis; therefore, the adjusted arrhythmia-type estimates cannot be interpreted as treatment-independent causal effects. The sensitivity analyses in Supplementary Table S1 yielded similar arrhythmia-type estimates across the examined model specifications; however, they cannot exclude unmeasured treatment-related confounding or selection bias.

Third, rhythm-control management was not identical between the two arrhythmia groups. Catheter ablation was performed in 83.7% of patients with AFL compared with 24.6% of those with AF, including ablation during the index hospitalization in 46.9% and 0%, respectively. This substantial imbalance reflects the established role of cavotricuspid isthmus ablation in typical AFL but may also have influenced subsequent arrhythmia burden and long-term outcomes. Because post-index ablation represents a treatment occurring after the start of follow-up, its appropriate incorporation into survival models would require time-dependent modeling. Exact dates of post-index ablation were not consistently available, precluding such an analysis. Consequently, residual confounding related to differences in rhythm-control management cannot be excluded, and the present findings should be interpreted as outcomes observed under the treatment strategies actually applied rather than as a comparison of the untreated natural history of AF and AFL. Although time-varying coefficients were used to address violations of the proportional hazards assumption, the observational nature of the study precludes complete elimination of residual confounding, and the time-dependent associations identified should not be interpreted as causal effects.

An exploratory sensitivity analysis excluding the 46 patients who underwent catheter ablation during the index hospitalization yielded adjusted AFL-versus-AF hazard ratios of 1.33 (95% CI 0.72–2.49; *p* = 0.37) for cardiovascular death and 0.85 (95% CI 0.41–1.77; *p* = 0.67) for first HF hospitalization (Supplementary Table S6). These estimates remained imprecise and shifted relative to the primary analysis; the restriction does not eliminate differences in subsequent ablation exposure and may introduce additional selection bias because index-hospitalization treatment was determined after study entry.

The marked between-group difference in catheter ablation exposure is a central limitation of the AF-versus-AFL comparison, rather than merely a difference in procedural practice. In particular, the observed long-term outcomes reflect prognosis according to the index arrhythmia type under the substantially different treatment pathways received during follow-up. Because post-index treatment could influence subsequent arrhythmia burden and clinical outcomes, these analyses cannot isolate the intrinsic prognostic contribution of AF versus AFL independent of treatment. The absence of a statistically significant adjusted association should therefore not be interpreted as evidence of prognostic equivalence.

Non-cardiovascular death was additionally treated as a competing event for cardiovascular death. The Fine–Gray subdistribution HR for AFL versus AF was 1.07 (95% CI 0.63–1.83), close to the primary cause-specific extended Cox estimate (Supplementary Table S8). As with the HF hospitalization analysis, the subdistribution and cause-specific hazards address distinct estimands. The competing-risk sensitivity models used fixed baseline coefficients; wide confidence intervals and the observational design do not permit conclusions of equivalent intrinsic risks.

Death was also evaluated as a competing event for first HF hospitalization. The Fine–Gray analysis yielded an adjusted subdistribution HR of 0.70 (95% CI 0.38–1.30), broadly similar in magnitude to the primary cause-specific extended Cox estimate, although the two models address different estimands (Supplementary Table S8). The Fine–Gray result relates to the subdistribution hazard, which is linked to the cumulative incidence of HF hospitalization in the presence of competing death; it does not directly estimate a risk ratio or establish prognostic equivalence. This exploratory model used fixed baseline coefficients rather than the time-varying effects of the primary Cox model. Together with the limited number of HF events in the AFL group, these considerations warrant cautious interpretation.

The complexity of the adjusted survival models also warrants caution. The final extended Cox models estimated 11 coefficients for 96 cardiovascular deaths (approximately 8.7 events per coefficient) and 17 coefficients for 91 first HF hospitalizations (approximately 5.4 events per coefficient), counting the time-varying terms. Particularly for HF hospitalization, the relatively low number of events per fitted coefficient raises the possibility of overfitting and unstable estimates. Although the alternative covariate specifications, including the parsimonious HF model (Supplementary Table S1), yielded broadly similar AFL-versus-AF estimates, these checks cannot exclude model instability or residual confounding. These adjusted findings should therefore be interpreted cautiously.

The AFL subgroup was substantially smaller than the AF subgroup (98 vs. 558 patients), with only 18 cardiovascular deaths and 13 HF hospitalizations among patients presenting with AFL. Consequently, the adjusted estimates for AFL versus AF were imprecise (cardiovascular death: aHR 1.09, 95% CI 0.64–1.85; HF hospitalization: aHR 0.69, 95% CI 0.37–1.27), and the confidence intervals remain compatible with potentially clinically meaningful differences in either direction, depending on the outcome. The absence of statistical significance should therefore not be interpreted as evidence of equivalence or non-inferiority between AF and AFL. The limited number of events in the AFL group also restricts the strength of subgroup-specific conclusions.

In analyses additionally adjusted for the calendar year of index TEE, the AFL-versus-AF estimates for LAAT, cardiovascular death, and HF hospitalization remained similar to the primary estimates (Supplementary Table S5). Nevertheless, adjustment for enrollment year cannot fully account for changes in treatment patterns, unmeasured calendar-time confounding, or differences in follow-up duration across recruitment years.

The number of patients with AFL remaining at risk decreased substantially over time (26 and 22 patients at 8 years for cardiovascular death and HF hospitalization, respectively, and 6 and 5 patients at 10 years, respectively). Accordingly, the late portions of the Kaplan–Meier curves should be interpreted cautiously. For graphical clarity, Figure 1 and Figure 2 are restricted to 8 years, while survival analyses use the full available follow-up.

Fourth, the prolonged recruitment and follow-up period spanned substantial changes in cardiovascular care. During this time, international guideline recommendations were repeatedly updated, DOACs became increasingly used, rhythm-control strategies evolved, and the management of major comorbidities—particularly HF—changed with the introduction and broader implementation of evidence-based therapies. Although the predefined anticoagulation eligibility criteria and TEE-based diagnostic pathway remained consistent, these secular changes in treatment could have influenced long-term cardiovascular outcomes and introduced calendar-time heterogeneity that may not be fully captured by adjustment for enrollment year.

HF at baseline was identified from previously documented clinical diagnoses rather than classified according to contemporary LVEF-based phenotypes. Consequently, this variable may encompass patients with reduced, mildly reduced, or preserved LVEF, and phenotype-specific associations could not be reliably assessed.

Similarly, CHA_2_DS_2_-VA was applied retrospectively as a standardized analytic variable rather than as a representation of the risk score used in clinical decision-making at enrollment. Changes in guideline-recommended risk assessment over the recruitment period should be considered when interpreting this historical cohort.

Fifth, anticoagulation adequacy was assessed using serial INR measurements in VKA-treated patients, whereas DOAC adherence before the index TEE was based on patient report and could not be objectively verified. This may have resulted in misclassification of anticoagulant exposure and should be considered when interpreting LAAT prevalence among patients meeting the study-defined anticoagulation criteria. In addition, the presence or absence of LAAT was determined by TEE and was not independently verified using an alternative imaging modality, such as contrast-enhanced echocardiography or cardiac computed tomography. Thus, the observed LAAT prevalence should be interpreted as occurring among patients who met the study-defined anticoagulation criteria, not necessarily among patients with independently confirmed uninterrupted therapeutic anticoagulant exposure. Bleeding events were not systematically recorded during follow-up; therefore, bleeding outcomes and the safety of the anticoagulation regimens could not be assessed in this cohort. Detailed longitudinal information on temporary anticoagulant interruptions, dose modifications, switching between agents, and treatment adherence was not systematically collected. Thus, baseline anticoagulation status could not establish continuous therapeutic anticoagulant exposure throughout follow-up.

In addition, the predefined dose-reduction eligibility criteria used in this historical cohort did not necessarily correspond to regulatory product-label criteria, particularly for rivaroxaban when a HAS-BLED score ≥ 3 was used as a reason for dose reduction. Renal function was assessed using MDRD-derived eGFR rather than Cockcroft–Gault creatinine clearance, limiting retrospective verification of product-label dosing appropriateness. Consequently, potential effects of non-standard dose selection on LAAT prevalence and long-term outcomes cannot be excluded. Individual-level data required to retrospectively calculate Cockcroft–Gault creatinine clearance were unavailable. Therefore, dosing appropriateness could not be comprehensively verified, and a sensitivity analysis restricted to patients with confirmed appropriate DOAC dosing was not feasible.

Clinical outcomes were ascertained using hospital discharge diagnoses, available medical documentation, and national healthcare databases with nationwide hospitalization coverage, supplemented by contact with patients or their relatives. Outcomes were classified according to a predefined scheme. Although formal independent endpoint adjudication and blinded outcome assessment were not performed, these complementary sources supported long-term outcome ascertainment. Nevertheless, some degree of outcome misclassification cannot be entirely excluded. Ischemic stroke, TIA, MI, and STE were analyzed descriptively and were not included in the adjusted time-to-event analyses performed for cardiovascular death and HF hospitalization.

Finally, the findings may not be generalizable to patients with newly diagnosed or untreated arrhythmias, asymptomatic patients, patients not meeting the predefined anticoagulation eligibility criteria, or those managed exclusively with a rate-control strategy.

## 5. Conclusions

In this prospective observational cohort of anticoagulated patients with AF or AFL referred for TEE-guided rhythm control, no statistically significant independent association was detected between index arrhythmia type (AF versus AFL) and LAAT, cardiovascular death, or HF hospitalization. However, catheter-ablation exposure differed markedly between the groups, and the observed long-term outcomes reflect the different rhythm-control treatment pathways actually applied. The observational design, residual confounding related to treatment, and imprecision of the estimates preclude conclusions regarding equivalent intrinsic prognoses for AF and AFL. Furthermore, the absence of systematic rhythm surveillance means that subsequent AF in patients presenting with AFL could not be reliably identified; the findings concern index arrhythmia classification rather than a stable long-term rhythm phenotype. No conclusions about comparative risks of ischemic stroke, TIA, MI, STE, or all-cause mortality can be inferred from the absence of a statistically significant adjusted association for cardiovascular death or HF hospitalization. These findings should be interpreted within the context of this selected population and warrant confirmation in larger studies.

## Figures and Tables

**Table 1:** Eligibility criteria for study enrollment.

Category	Inclusion Criteria	Exclusion Criteria
Age	≥18 years	–
Arrhythmia	Atrial fibrillation (AF) cohort: symptomatic AF lasting > 48 h referred for a planned rhythm-control strategy. Atrial flutter (AFL) cohort: symptomatic AFL lasting > 48 h referred for a planned rhythm-control strategy.	–
Anticoagulation	Meeting predefined anticoagulation eligibility criteria	–
Previous procedures	–	Previous electrical cardioversion; Previous catheter ablation for AF or AFL
Structural heart disease	–	Previous intracardiac thrombus; Prosthetic heart valve; Moderate or severe mitral stenosis (mitral valve area ≤ 1.5 cm^2^ with a mean gradient ≥ 5 mmHg)
Clinical status	–	Heart rate < 60 beats/min at admission; Systolic blood pressure < 90 mmHg; Symptoms or signs of acute heart failure exacerbation; Symptoms or signs of peripheral hypoperfusion

**Table 2:** Clinical characteristics, treatment, and outcomes according to index arrhythmia type.

Variable	AF (*n* = 558)	AFL (*n* = 98)	*p* Value	SMD
Clinical and echocardiographic characteristics				
Age, years	64.9 (9.8)	68.4 (10.9)	**0.001**	0.34
Male sex	335 (60.0%)	59 (60.2%)	0.98	0.00
BMI, kg/m^2^	29.3 (27–32.8)	29.1 (27.5–33.4)	0.60	0.06
Systolic blood pressure, mm Hg	123.9 (10.8)	122.2 (12.0)	0.16	0.15
Diastolic blood pressure, mm Hg	77.2 (7.5)	77.7 (8.0)	0.57	0.06
Heart rate, beats/min	90 (80–110)	114.5 (84.2–130.8)	**<0.001**	0.61
eGFR, mL/min/1.73 m^2^	67.3 (15.3)	64.1 (15.8)	0.06	0.21
Left atrial diameter, mm	44.8 (5.0)	43.9 (5.7)	0.12	0.17
LVEF, %	57 (50–60)	55 (45–60)	0.13	0.17
CHA_2_DS_2_-VA score	3 (2–3)	3 (2–4)	**0.001**	0.28
Age ≥ 75 years	82 (14.7%)	29 (29.6%)	**<0.001**	0.36
Left atrial diameter > 40 mm	461 (82.6%)	70 (71.4%)	**0.009**	0.27
LVEF < 40%	64 (11.5%)	14 (14.3%)	0.43	0.08
Medical history				
Hypertension	435 (78.0%)	83 (84.7%)	0.13	0.17
History of heart failure	326 (58.4%)	45 (45.9%)	**0.02**	0.25
Diabetes mellitus	130 (23.3%)	35 (35.7%)	**0.009**	0.27
Previous myocardial infarction	40 (7.2%)	12 (12.2%)	0.09	0.17
Previous stroke/TIA/systemic thromboembolism	41 (7.3%)	9 (9.2%)	0.53	0.07
Peripheral artery disease	8 (1.4%)	1 (1.0%)	0.99	0.04
Aortic atherosclerosis	37 (6.6%)	10 (10.2%)	0.21	0.13
Chronic obstructive pulmonary disease	25 (4.5%)	4 (4.1%)	0.99	0.02
Index TEE and pharmacological treatment				
Left atrial appendage thrombus	79 (14.2%)	10 (10.2%)	0.29	0.12
VKA on admission	64 (11.5%)	10 (10.2%)	0.72	0.04
Rivaroxaban on admission	161 (28.9%)	31 (31.6%)	0.58	0.06
Apixaban on admission	97 (17.4%)	14 (14.3%)	0.45	0.08
Dabigatran on admission	236 (42.3%)	43 (43.9%)	0.77	0.03
VKA at discharge	63 (11.3%)	13 (13.3%)	0.57	
Rivaroxaban at discharge	154 (27.6%)	30 (30.6%)	0.54	
Apixaban at discharge	104 (18.6%)	16 (16.3%)	0.59	
Dabigatran at discharge	236 (42.3%)	39 (39.8%)	0.64	
ACE inhibitor/ARB at discharge	408 (73.1%)	68 (69.4%)	0.45	
Beta-blocker at discharge	494 (88.5%)	85 (86.7%)	0.61	
Statin at discharge	354 (63.4%)	61 (62.2%)	0.82	
Rhythm-control treatment				
Electrical cardioversion during index hospitalization	462 (82.8%)	41 (41.8%)	**<0.001**	
Catheter ablation during index hospitalization	0 (0%)	46 (46.9%)	**<0.001**	
Catheter ablation after index hospitalization	137 (24.6%)	48 (49.0%)	**<0.001**	
Catheter ablation during the study period	137 (24.6%)	82 (83.7%)	**<0.001**	
Clinical outcomes				
Heart failure hospitalization	78 (14.0%)	13 (13.3%)	0.85	
Myocardial infarction	14 (2.5%)	2 (2.0%)	0.99	
Systemic thromboembolism	3 (0.5%)	0 (0%)	0.99	
Transient ischemic attack	6 (1.1%)	1 (1.0%)	0.99	
Stroke	17 (3.0%)	4 (4.1%)	0.54	
Cardiovascular death	78 (14.0%)	18 (18.4%)	0.26	
All-cause death	88 (15.8%)	23 (23.5%)	0.06	

SMD, standardized mean difference (absolute value). SMDs were calculated from group means and standard deviations for continuous variables and from exact counts for binary variables. SMDs describe baseline characteristics only; no SMD was calculated for discharge treatments, rhythm-control procedures, or follow-up outcomes. Minor differences may arise from rounding of continuous summary statistics. Data are presented as mean (SD) for approximately symmetric continuous variables, median (IQR) for skewed continuous variables and the CHA_2_DS_2_-VA score, and number (percentage) for categorical variables. Between-group comparisons used Student’s *t* test (age, systolic and diastolic blood pressure, eGFR), Welch’s *t* test (BMI, heart rate, left atrial diameter, LVEF), or the Mann–Whitney U test (CHA_2_DS_2_-VA score). For skewed variables summarized as median (IQR), Welch’s test compares means rather than medians. Categorical variables were compared using Pearson’s χ^2^ or Fisher’s exact test, as appropriate. Absolute SMDs for continuous variables were calculated from group means and SDs, regardless of the displayed summary measure. *p* values compare AF with AFL; *p* < 0.05 was considered statistically significant. Statistically significant *p* values are indicated in bold. Abbreviations: ACE, angiotensin-converting enzyme; AF, atrial fibrillation; AFL, atrial flutter; ARB, angiotensin receptor blocker; BMI, body mass index; CHA_2_DS_2_-VA, clinical thromboembolic risk score without the sex category; eGFR, estimated glomerular filtration rate; IQR, interquartile range; LVEF, left ventricular ejection fraction; SD, standard deviation; SMD, standardized mean difference; TEE, transesophageal echocardiography; TIA, transient ischemic attack; and VKA, vitamin K antagonist.

**Table 3:** Univariable and multivariable logistic regression analyses for LAAT.

Variable	Univariable OR (95% CI)	*p* Value	Multivariable aOR (95% CI)	*p* Value
Index arrhythmia type (AFL vs. AF)	0.69 (0.34–1.38)	0.29	0.53 (0.25–1.11)	0.09
LVEF, per 1% increase	0.94 (0.93–0.96)	**<0.001**	0.95 (0.93–0.97)	**<0.001**
Left atrial diameter, per 1 mm increase	1.10 (1.06–1.15)	**<0.001**	1.04 (0.99–1.09)	0.15
eGFR, per 1 mL/min/1.73 m^2^ increase	0.98 (0.97–1.00)	**0.04**	1.00 (0.98–1.02)	0.90
CHA_2_DS_2_-VA score, per 1-point increase	1.47 (1.26–1.73)	**<0.001**	1.41 (1.18–1.69)	**<0.001**

AF was the reference category for index arrhythmia type. Index arrhythmia type was included a priori in the multivariable model, irrespective of its statistical significance in univariable analysis. Bold values indicate statistically significant associations (*p* < 0.05). Abbreviations: AF, atrial fibrillation; AFL, atrial flutter; aOR, adjusted odds ratio; CI, confidence interval; eGFR, estimated glomerular filtration rate; CHA_2_DS_2_-VA, clinical thromboembolic risk score without the sex category; LAAT, left atrial appendage thrombus; LVEF, left ventricular ejection fraction; OR, odds ratio.

**Table 4:** Multivariable conventional and extended Cox regression analyses of cardiovascular death and HF hospitalization. Panel (**A**). Multivariable adjusted hazard ratios in the final extended Cox models; Panel (**B**). Time-varying coefficients in the final extended Cox models; Panel (**C**). Model fit: conventional vs. extended Cox model.

(**A**)				
Variable	Cardiovascular Death aHR (95% CI)	*p* Value	HF Hospitalization aHR (95% CI)	*p* Value
Index arrhythmia type (AFL vs. AF)	1.09 (0.64–1.85)	0.75	0.69 (0.37–1.27)	0.23
LAAT	1.71 (1.04–2.81)	**0.03**	1.58 (0.94–2.66)	0.08
Age, per 1-year increase	1.03 (0.99–1.06)	0.11	1.02 (0.98–1.06)	0.36
CHA_2_DS_2_-VA score, per 1-point increase	1.08 (0.88–1.33)	0.46	1.06 (0.74–1.52)	0.75
Heart rate, per 1 beat/min increase	–	–	1.01 (1.00–1.02)	0.06
eGFR, per 1 mL/min/1.73 m^2^ increase	0.99 (0.97–1.00)	0.12	0.97 (0.96–0.99)	**0.002**
Left atrial diameter, per 1 mm increase	1.04 (1.00–1.09)	0.07	1.10 (1.06–1.15)	**<0.001**
LVEF, per 1% increase	0.97 (0.96–0.99)	**0.003**	Time-varying effect *	–
Previous stroke/TIA/systemic thromboembolism	–	–	Time-varying effect *	–
History of HF	Time-varying effect *	–	Time-varying effect *	–
Diabetes mellitus	–	–	1.36 (0.74–2.52)	0.32
Previous myocardial infarction	1.58 (0.88–2.83)	0.13	1.85 (0.97–3.54)	0.06
Peripheral artery disease	4.56 (1.51–13.81)	**0.007**	5.81 (1.60–21.06)	**0.008**
VKA on admission	–	–	1.42 (0.85–2.38)	0.18
(**B**)				
Time-varying term	Cardiovascular death HR (95% CI)	*p* value	HF hospitalization HR (95% CI)	*p* value
History of HF—main coefficient β	346.340 (1.870–64,157.416) β = +5.847	**0.03**	6.648 (0.447–98.828) β = +1.894	0.17
History of HF × log(time)	0.452 (0.220–0.929) γ = −0.793	**0.03**	0.717 (0.475–1.083) γ = −0.333	0.11
LVEF—main coefficient β	–	–	0.934 (0.876–0.995) β = −0.069	**0.04**
LVEF × log(time)	–	–	1.007 (0.996–1.018) γ = +0.007	0.21
Previous stroke/TIA/systemic thromboembolism—main coefficient β	–	–	0.032 (0.001–1.572) β = −3.439	0.08
Previous stroke/TIA/systemic thromboembolism × log(time)	–	–	1.825 (1.038–3.208) γ = +0.601	**0.04**
(**C**)				
Criterion	Cardiovascular death		HF hospitalization	
Likelihood-ratio test	χ^2^ = 6.24; df = 1; ***p* = 0.01**		χ^2^ = 15.04; df = 3; ***p* = 0.002**	
AIC, conventional model	1093.90		1011.29	
AIC, extended model	1089.66		1002.25	
ΔAIC	−4.24		−9.04	

**Notes.** Table 4 presents adjusted estimates only; corresponding univariable Cox regression results are provided in Supplementary Table S2. Conventional Cox models assume proportional hazards, whereas extended Cox models include time-varying coefficients for covariates violating this assumption. Main-effect and time-varying coefficients in extended models must be interpreted jointly; a main-effect HR alone should not be interpreted as constant throughout follow-up. Panel (**B**) reports both the main coefficient (β) and the time-interaction coefficient (γ) for each covariate with a time-varying effect. HR(t) = exp[β + γ × ln(t)], where t is uncentered follow-up time in days. Consequently, exp(β) corresponds to t = 1 day and must not be interpreted as a constant HR. For γ rows, the exponentiated interaction coefficient, its 95% CI, and *p* value are retained. Time-specific HRs and 95% CIs at 1, 3, 5, and 8 years are reported in Supplementary Table S7. * In the final extended Cox models, history of HF (cardiovascular death) and history of HF, LVEF, and previous stroke/TIA/systemic thromboembolism (HF hospitalization) were modeled with time-varying coefficients because the proportional hazards assumption was violated. Their main and time-varying coefficients jointly define HR(t) and should not be interpreted as a single constant hazard ratio. Multivariable estimates shown for covariates satisfying the proportional hazards assumption are from the final extended Cox models. Variables marked with a dash were not included in the respective multivariable model. Reference categories for binary variables were: AF for index arrhythmia type; and absence/no for clinical conditions and drug exposure. Bold values indicate statistically significant associations (*p* < 0.05). Abbreviations: AF, atrial fibrillation; AFL, atrial flutter; AIC, Akaike information criterion; CHA_2_DS_2_-VA, clinical thromboembolic risk score without the sex category; CI, confidence interval; eGFR, estimated glomerular filtration rate; HF, heart failure; LAAT, left atrial appendage thrombus; LVEF, left ventricular ejection fraction; TIA, transient ischemic attack; and VKA, vitamin K antagonist.

## Data Availability

Data supporting this study are available from the corresponding author, Ł.T., upon reasonable request.

## Supplementary Materials

The following supporting information can be downloaded at: https://www.mdpi.com/article/10.3390/jcm15218552/s1, Table S1: Sensitivity analyses for cardiovascular death and HF hospitalization. Table S2: Univariable Cox regression analyses for cardiovascular death and HF hospitalization. Table S3: Prevalence of LAAT by anticoagulant at admission and index arrhythmia type. Table S4: Schoenfeld residual-based proportional hazards diagnostics and time-varying coefficients for the primary multivariable Cox models of cardiovascular death and HF hospitalization. Table S5: Sensitivity analyses additionally adjusted for calendar year of index TEE. Table S6: Exploratory sensitivity analyses excluding index-hospitalization catheter ablation. Table S7: Time-specific adjusted hazard ratios for covariates with time-varying coefficients in the extended Cox models. Table S8: Competing-risk sensitivity analyses for cardiovascular death and first HF hospitalization (AFL versus AF). Table S9: Variance inflation factors (VIFs) for baseline covariates in the primary survival models.

## Abbreviations

The following abbreviations are used in this manuscript:

ACE	Angiotensin-converting enzyme
AF	Atrial fibrillation
AFL	Atrial flutter
aHR	Adjusted hazard ratio
aOR	Adjusted odds ratio
ARB	Angiotensin receptor blocker
BMI	Body mass index
CHA_2_DS_2_-VA	Clinical thromboembolic risk score without the sex category
CHF	Congestive heart failure
CI	Confidence interval
CV	Cardiovascular
DOAC	Direct oral anticoagulant
eGFR	Estimated glomerular filtration rate
HF	Heart failure
HR	Hazard ratio
INR	International normalized ratio
IQR	Interquartile range
LAAT	Left atrial appendage thrombus
LRT	Likelihood ratio test
LVEF	Left ventricular ejection fraction
MDRD	Modification of Diet in Renal Disease
OR	Odds ratio
PH	Proportional hazards
SD	Standard deviation
sHR	Subdistribution hazard ratio
SMD	Standardized mean difference
STE	Systemic thromboembolism
TEE	Transesophageal echocardiography
TIA	Transient ischemic attack
VIF	Variance inflation factor
VKA	Vitamin K antagonist
