# Supplementary material for: Left Atrial Appendage Thrombus and Long-Term Clinical Outcomes in Anticoagulated Patients with Atrial Fibrillation and Atrial Flutter Referred for a Rhythm-Control Strategy: A Prospective Observational Cohort Study
Source: J Clin Med. 2026 Nov 3;15(21):8552. doi: 10.3390/jcm15218552 (PMC13763755; doi:10.3390/jcm15218552)
Supplement: Supplementary file 1 [file jcm-15-08552-s001.zip › AF_AFL_Table_S1_JCM.pdf]

Supplementary Materials

Left Atrial Appendage Thrombus and Long-Term Clinical Outcomes in Anticoagulated Patients With Atrial Fibrillation and Atrial Flutter Referred for a Rhythm-Control Strategy: A Prospective Observational Cohort Study

Łukasz Turek, Marcin Sadowski, Łukasz Zandecki, Jacek Kurzawski, Marianna Janion

Table S1. Sensitivity analyses for cardiovascular death and HF hospitalization.

Outcome	Model specification	AFL vs AF, aHR (95% CI)	*p* value
Cardiovascular death	Full extended Cox model (primary analysis)	1.09 (0.64–1.85)	0.75
Cardiovascular death	Model excluding CHA_2DS_2-VA score	1.15 (0.68–1.96)	0.61
HF hospitalization	Full extended Cox model (primary analysis)	0.69 (0.37–1.27)	0.23
HF hospitalization	Parsimonious multivariable model	0.76 (0.42–1.40)	0.38
HF hospitalization	Model excluding CHA_2DS_2-VA score	0.69 (0.37–1.28)	0.24

Note: The full extended Cox models are presented for comparison with the sensitivity analyses, which assessed the robustness of the association between the index arrhythmia type (AF versus AFL) and the principal time-to-event outcomes across alternative model specifications. The index arrhythmia type (AFL vs AF) was retained in all models irrespective of statistical significance. Non-proportional hazards were addressed using time-varying coefficients where required. The parsimonious multivariable Cox model for HF hospitalization included 10 baseline covariates: index arrhythmia type (AFL versus AF), left ventricular ejection fraction (LVEF), left atrial diameter, estimated glomerular filtration rate (eGFR; MDRD), left atrial appendage thrombus (LAAT), CHA_2DS_2-VA score, age, history of myocardial infarction, history of heart failure, and vitamin K antagonist (VKA) use on admission. The model additionally included time-varying coefficient terms for LVEF [tt(LVEF)] and history of HF [tt(history of HF)]. The model excluding CHA_2DS_2-VA was examined as a sensitivity analysis to assess whether the principal exposure estimate was influenced by potential redundancy or collinearity between the composite risk score and its related individual clinical covariates. AF was the reference category.

Abbreviations: AF, atrial fibrillation; AFL, atrial flutter; aHR, adjusted hazard ratio; CHA_2DS_2-VA, clinical thromboembolic risk score without the sex category; CI, confidence interval; eGFR, estimated glomerular filtration rate; HF, heart failure; LAAT, left atrial appendage thrombus; LVEF, left ventricular ejection fraction; MDRD, Modification of Diet in Renal Disease; VKA, vitamin K antagonist.
