# Supplementary material for: Left Atrial Appendage Thrombus and Long-Term Clinical Outcomes in Anticoagulated Patients with Atrial Fibrillation and Atrial Flutter Referred for a Rhythm-Control Strategy: A Prospective Observational Cohort Study
Source: J Clin Med. 2026 Nov 3;15(21):8552. doi: 10.3390/jcm15218552 (PMC13763755; doi:10.3390/jcm15218552)
Supplement: Supplementary file 1 [file jcm-15-08552-s001.zip › AF_AFL_Table_S2_JCM.pdf]

# Supplementary Materials

Left Atrial Appendage Thrombus and Long-Term Clinical Outcomes in Anticoagulated Patients With Atrial Fibrillation and Atrial Flutter Referred for a Rhythm-Control Strategy: A Prospective Observational Cohort Study

Łukasz Turek, Marcin Sadowski, Łukasz Zandecki, Jacek Kurzawski, Marianna Janion

**Table S2.** Univariable Cox regression analyses for cardiovascular death and HF hospitalization.

| Variable | Cardiovascular death HR (95% CI) | *p* value | HF hospitalization HR (95% CI) | *p* value |
|---|---|---|---|---|
| Index arrhythmia type (AFL vs AF) | 1.23 (0.74–2.06) | 0.42 | 0.90 (0.50–1.62) | 0.73 |
| Left atrial appendage thrombus | 3.01 (1.92–4.73) | <0.001 | 3.53 (2.24–5.55) | <0.001 |
| Male sex | 1.18 (0.77–1.79) | 0.44 | 0.99 (0.65–1.51) | 0.96 |
| Age, per 1-year increase | 1.05 (1.03–1.07) | <0.001 | 1.04 (1.02–1.07) | <0.001 |
| BMI, per 1 kg/m² increase | 0.96 (0.91–1.00) | 0.07 | 1.02 (0.98–1.07) | 0.35 |
| $CHA_2DS_2$-VA score, per 1-point increase | 1.44 (1.26–1.65) | <0.001 | 1.45 (1.26–1.67) | <0.001 |
| Heart rate, per 1 beat/min increase | 1.01 (1.00–1.02) | 0.20 | 1.01 (1.00–1.02) | 0.02 |
| Systolic blood pressure, per 1 mm Hg increase | 0.99 (0.97–1.01) | 0.34 | 1.00 (0.98–1.02) | 0.98 |
| Diastolic blood pressure, per 1 mm Hg increase | 0.99 (0.97–1.02) | 0.65 | 1.02 (0.99–1.05) | 0.15 |
| eGFR, per 1 mL/min/1.73 m² increase | 0.97 (0.96–0.99) | <0.001 | 0.96 (0.95–0.97) | <0.001 |
| Left atrial diameter, per 1 mm increase | 1.09 (1.05–1.13) | <0.001 | 1.14 (1.10–1.19) | <0.001 |
| LVEF, per 1% increase | 0.95 (0.94–0.97) | <0.001 | 0.95 (0.93–0.96) | <0.001 |
| Previous stroke/TIA/systemic thromboembolism | 1.43 (0.74–2.74) | 0.29 | 1.93 (1.05–3.55) | 0.03 |
| Hypertension | 1.21 (0.72–2.02) | 0.47 | 1.61 (0.91–2.84) | 0.10 |
| History of heart failure | 2.18 (1.43–3.31) | <0.001 | 1.80 (1.17–2.77) | 0.008 |
| Diabetes mellitus | 1.36 (0.87–2.14) | 0.18 | 1.58 (1.01–2.47) | 0.047 |
| Chronic obstructive pulmonary disease | 1.47 (0.64–3.36) | 0.36 | 1.01 (0.37–2.75) | 0.99 |
| Previous myocardial infarction | 2.31 (1.33–4.01) | 0.003 | 2.32 (1.31–4.11) | 0.004 |
| Peripheral artery disease | 5.20 (1.90–14.21) | 0.001 | 4.01 (1.27–12.72) | 0.02 |
| VKA on admission | 1.64 (1.01–2.65) | 0.046 | 1.89 (1.15–3.11) | 0.01 |
| Rivaroxaban on admission | 0.86 (0.54–1.37) | 0.53 | 1.08 (0.69–1.69) | 0.74 |
| Apixaban on admission | 1.04 (0.53–2.02) | 0.92 | 0.78 (0.39–1.58) | 0.50 |
| Dabigatran on admission | 0.82 (0.55–1.23) | 0.34 | 0.71 (0.46–1.08) | 0.11 |
| VKA at discharge | 1.46 (0.89–2.38) | 0.13 | 1.55 (0.92–2.59) | 0.10 |
| Rivaroxaban at discharge | 0.75 (0.46–1.21) | 0.24 | 1.13 (0.72–1.77) | 0.60 |
| Apixaban at discharge | 0.98 (0.52–1.86) | 0.96 | 1.07 (0.59–1.95) | 0.82 |
| Dabigatran at discharge | 1.00 (0.67–1.49) | 0.99 | 0.68 (0.44–1.04) | 0.08 |
| ACE inhibitor/ARB at discharge | 1.38 (0.85–2.24) | 0.19 | 1.16 (0.72–1.86) | 0.54 |

| | | | | |
|---|---|---|---|---|
| Beta-blocker at discharge | 2.46 (1.00–6.06) | 0.0504 | 2.05 (0.89–4.69) | 0.09 |
| Statin at discharge | 0.87 (0.58–1.31) | 0.51 | 0.92 (0.60–1.40) | 0.68 |

Note: Hazard ratios were estimated using separate univariable Cox regression models and are unadjusted. AF was the reference category for the comparison of index arrhythmia types. Hazard ratios for continuous variables are expressed per unit increase, as indicated in the table.
Abbreviations: ACE, angiotensin-converting enzyme; AF, atrial fibrillation; AFL, atrial flutter; ARB, angiotensin receptor blocker; BMI, body mass index; $CHA_2DS_2$-VA, clinical thromboembolic risk score without the sex category; CI, confidence interval; eGFR, estimated glomerular filtration rate; HF, heart failure; HR, hazard ratio; LVEF, left ventricular ejection fraction; TIA, transient ischemic attack; VKA, vitamin K antagonist.
