# Supplementary material for: Left Atrial Appendage Thrombus and Long-Term Clinical Outcomes in Anticoagulated Patients with Atrial Fibrillation and Atrial Flutter Referred for a Rhythm-Control Strategy: A Prospective Observational Cohort Study
Source: J Clin Med. 2026 Nov 3;15(21):8552. doi: 10.3390/jcm15218552 (PMC13763755; doi:10.3390/jcm15218552)
Supplement: Supplementary file 1 [file jcm-15-08552-s001.zip › AF_AFL_Table_S3_JCM.pdf]

# Supplementary Materials

Left Atrial Appendage Thrombus and Long-Term Clinical Outcomes in Anticoagulated Patients With Atrial Fibrillation and Atrial Flutter Referred for a Rhythm-Control Strategy: A Prospective Observational Cohort Study

Łukasz Turek, Marcin Sadowski, Łukasz Zandecki, Jacek Kurzawski, Marianna Janion

**Table S3.** Prevalence of LAAT by anticoagulant at admission and index arrhythmia type.

| Anticoagulant at admission | Overall (N = 656) | AF (N = 558) | AFL (N = 98) |
|---|---|---|---|
| VKA | 14/74 (18.9%) | 14/64 (21.9%) | 0/10 (0.0%) |
| Dabigatran | 38/279 (13.6%) | 31/236 (13.1%) | 7/43 (16.3%) |
| Rivaroxaban | 22/192 (11.5%) | 20/161 (12.4%) | 2/31 (6.5%) |
| Apixaban | 15/111 (13.5%) | 14/97 (14.4%) | 1/14 (7.1%) |
| All DOACs | 75/582 (12.9%) | 65/494 (13.2%) | 10/88 (11.4%) |
| Total | 89/656 (13.6%) | 79/558 (14.2%) | 10/98 (10.2%) |

Note: Values are presented as n/N (%), where n is the number of patients with LAAT and N is the number receiving the specified anticoagulant at index admission. The analysis is descriptive and unadjusted; small subgroup sizes, particularly among VKA-treated patients with AFL, preclude conclusions about comparative anticoagulant effectiveness. Uninterrupted DOAC use during the preceding 3 weeks was assessed by patient declaration, whereas VKA eligibility required three consecutive weekly INR measurements of at least 2.0.

Abbreviations: AF, atrial fibrillation; AFL, atrial flutter; DOAC, direct oral anticoagulant; INR, international normalized ratio; LAAT, left atrial appendage thrombus; VKA, vitamin K antagonist.
