# Supplementary material for: Left Atrial Appendage Thrombus and Long-Term Clinical Outcomes in Anticoagulated Patients with Atrial Fibrillation and Atrial Flutter Referred for a Rhythm-Control Strategy: A Prospective Observational Cohort Study
Source: J Clin Med. 2026 Nov 3;15(21):8552. doi: 10.3390/jcm15218552 (PMC13763755; doi:10.3390/jcm15218552)
Supplement: Supplementary file 1 [file jcm-15-08552-s001.zip › AF_AFL_Table_S4_JCM.pdf]

# Supplementary Materials

Left Atrial Appendage Thrombus and Long-Term Clinical Outcomes in Anticoagulated Patients With Atrial Fibrillation and Atrial Flutter Referred for a Rhythm-Control Strategy: A Prospective Observational Cohort Study

Łukasz Turek, Marcin Sadowski, Łukasz Zandecki, Jacek Kurzawski, Marianna Janion

**Table S4.** Schoenfeld residual-based proportional hazards diagnostics and time-varying coefficients for the primary multivariable Cox models of cardiovascular death and HF hospitalization.

| Panel A. Proportional hazards diagnostics for cardiovascular death | | | |
|---|---|---|---|
| Covariate | Schoenfeld $\chi^2$ | df | *p* value |
| Index arrhythmia type (AFL vs AF) | 1.58 | 1 | 0.21 |
| LVEF | 0.83 | 1 | 0.36 |
| $CHA_2DS_2$-VA | 2.08 | 1 | 0.15 |
| LAAT | 1.95 | 1 | 0.16 |
| Left atrial diameter (mm) | 1.29 | 1 | 0.26 |
| Age (years) | 0.61 | 1 | 0.44 |
| eGFR using the MDRD (mL/min/1.73 $m^2$) | 0.01 | 1 | 0.92 |
| History of HF | 4.43 | 1 | 0.04 |
| Peripheral artery disease | 0.63 | 1 | 0.43 |
| Myocardial infarction | 0.28 | 1 | 0.60 |
| GLOBAL | 13.66 | 10 | 0.19 |

| Panel B. Proportional hazards diagnostics for HF hospitalization | | | |
|---|---|---|---|
| Covariate | Schoenfeld $\chi^2$ | df | *p* value |
| LAAT | 0.70 | 1 | 0.40 |
| Age (years) | 2.04 | 1 | 0.15 |
| $CHA_2DS_2$-VA | 0.73 | 1 | 0.39 |
| Heart rate (beats/min) | 0.56 | 1 | 0.46 |
| eGFR using the MDRD (mL/min/1.73 $m^2$) | 0.09 | 1 | 0.77 |
| Left atrial diameter (mm) | 3.79 | 1 | 0.051 |
| LVEF | 9.85 | 1 | 0.002 |
| Previous stroke/TIA/systemic thromboembolism | 6.23 | 1 | 0.01 |
| History of HF | 7.55 | 1 | 0.006 |
| Diabetes mellitus | 2.87 | 1 | 0.09 |
| Myocardial infarction | 1.57 | 1 | 0.21 |
| Peripheral artery disease | 2.63 | 1 | 0.10 |
| VKA on admission | 1.31 | 1 | 0.25 |
| Index arrhythmia type (AFL vs AF) | 2.00 | 1 | 0.16 |

| | | | |
|---|---|---|---|
| GLOBAL | 29.57 | 14 | 0.009 |

| Panel C. Time-varying coefficients in the extended Cox models | | | | |
|---|---|---|---|---|
| Outcome | Covariate | γ for X × log(t) | exp(γ) (95% CI) | *p* value |
| CV death | History of HF | −0.793 | 0.452 (0.220–0.929) | 0.03 |
| HF hospitalization | History of HF | −0.333 | 0.717 (0.475–1.083) | 0.11 |
| HF hospitalization | LVEF | +0.007 | 1.007 (0.996–1.018) | 0.21 |
| HF hospitalization | Previous stroke/TIA/systemic thromboembolism | +0.601 | 1.825 (1.038–3.208) | 0.04 |

Note: Proportional hazards diagnostics were obtained from the conventional multivariable Cox models corresponding to the primary analyses in Table 4. Covariate-specific and global tests are based on Schoenfeld residuals. The extended models incorporated time-varying coefficients for covariates with evidence of non-proportional hazards. The main and interaction terms must be interpreted jointly: $HR(t) = \exp[\beta + \gamma \log(t)]$, where t is follow-up time in days. The exponentiated interaction coefficient exp(γ) is not a hazard ratio at a specific follow-up time.

Abbreviations: AF, atrial fibrillation; AFL, atrial flutter; CI, confidence interval; CV, cardiovascular; eGFR, estimated glomerular filtration rate; HF, heart failure; HR, hazard ratio; LAAT, left atrial appendage thrombus; LVEF, left ventricular ejection fraction; MDRD, Modification of Diet in Renal Disease; PH, proportional hazards; TIA, transient ischemic attack; VKA, vitamin K antagonist.
