# Supplementary material for: Left Atrial Appendage Thrombus and Long-Term Clinical Outcomes in Anticoagulated Patients with Atrial Fibrillation and Atrial Flutter Referred for a Rhythm-Control Strategy: A Prospective Observational Cohort Study
Source: J Clin Med. 2026 Nov 3;15(21):8552. doi: 10.3390/jcm15218552 (PMC13763755; doi:10.3390/jcm15218552)
Supplement: Supplementary file 1 [file jcm-15-08552-s001.zip › AF_AFL_Table_S5_JCM.pdf]

Supplementary Materials

Left Atrial Appendage Thrombus and Long-Term Clinical Outcomes in Anticoagulated Patients With Atrial Fibrillation and Atrial Flutter Referred for a Rhythm-Control Strategy: A Prospective Observational Cohort Study

Łukasz Turek, Marcin Sadowski, Łukasz Zandecki, Jacek Kurzawski, Marianna Janion

Table S5. Sensitivity analyses additionally adjusted for calendar year of index TEE.

Outcome	Model specification	AFL vs AF, adjusted estimate (95% CI)	*p* value
LAAT	Primary multivariable logistic regression	aOR 0.53 (0.25–1.11)	0.09
LAAT	Primary model + index TEE year	aOR 0.54 (0.26–1.14)	0.11
Cardiovascular death	Full extended Cox model (primary analysis)	aHR 1.09 (0.64–1.85)	0.75
Cardiovascular death	Full extended Cox model + index TEE year	aHR 1.09 (0.64–1.85)	0.75
First HF hospitalization	Full extended Cox model (primary analysis)	aHR 0.69 (0.37–1.27)	0.23
First HF hospitalization	Full extended Cox model + index TEE year	aHR 0.68 (0.37–1.27)	0.23

Note: Index year was defined as the calendar year of the index TEE (2010–2024) and added as a continuous covariate to the respective primary multivariable model, with all original covariates and time-varying terms retained. The extended Cox model for cardiovascular death retained a time-varying effect for history of heart failure; the extended Cox model for first heart failure hospitalization retained time-varying effects for left ventricular ejection fraction, history of stroke/transient ischemic attack/systemic embolism, and history of heart failure. The estimates compare AFL with AF. Adjustment for index year did not materially change the estimates; residual confounding by secular changes in care cannot be excluded.

Abbreviations: AF, atrial fibrillation; AFL, atrial flutter; aHR, adjusted hazard ratio; aOR, adjusted odds ratio; CI, confidence interval; HF, heart failure; LAAT, left atrial appendage thrombus; TEE, transesophageal echocardiography.
