# Supplementary material for: Left Atrial Appendage Thrombus and Long-Term Clinical Outcomes in Anticoagulated Patients with Atrial Fibrillation and Atrial Flutter Referred for a Rhythm-Control Strategy: A Prospective Observational Cohort Study
Source: J Clin Med. 2026 Nov 3;15(21):8552. doi: 10.3390/jcm15218552 (PMC13763755; doi:10.3390/jcm15218552)
Supplement: Supplementary file 1 [file jcm-15-08552-s001.zip › AF_AFL_Table_S6_JCM.pdf]

Supplementary Materials

Left Atrial Appendage Thrombus and Long-Term Clinical Outcomes in Anticoagulated Patients With Atrial Fibrillation and Atrial Flutter Referred for a Rhythm-Control Strategy: A Prospective Observational Cohort Study

Łukasz Turek, Marcin Sadowski, Łukasz Zandecki, Jacek Kurzawski, Marianna Janion

Table S6. Exploratory sensitivity analyses excluding index-hospitalization catheter ablation.

Outcome	Model specification	AFL vs AF, adjusted estimate (95% CI)	*p* value
Cardiovascular death	Primary extended Cox model (all patients)	aHR 1.09 (0.64–1.85)	0.75
Cardiovascular death	Without index-hospitalization ablation (n = 610)	aHR 1.33 (0.72–2.49)	0.37
First HF hospitalization	Primary extended Cox model (all patients)	aHR 0.69 (0.37–1.27)	0.23
First HF hospitalization	Without index-hospitalization ablation (n = 610)	aHR 0.85 (0.41–1.77)	0.67

Note: Exploratory sensitivity analyses excluded patients who underwent catheter ablation during the index hospitalization (46 patients with AFL and no patients with AF). The same baseline covariates and time-varying coefficient specifications as in the primary extended Cox models were retained. Treatment selection occurred after study entry; these restricted analyses are subject to selection bias, residual confounding by subsequent ablation, and limited precision. The results do not estimate treatment-independent causal effects.

Abbreviations: AF, atrial fibrillation; AFL, atrial flutter; aHR, adjusted hazard ratio; CI, confidence interval; HF, heart failure.
