# Supplementary material for: Left Atrial Appendage Thrombus and Long-Term Clinical Outcomes in Anticoagulated Patients with Atrial Fibrillation and Atrial Flutter Referred for a Rhythm-Control Strategy: A Prospective Observational Cohort Study
Source: J Clin Med. 2026 Nov 3;15(21):8552. doi: 10.3390/jcm15218552 (PMC13763755; doi:10.3390/jcm15218552)
Supplement: Supplementary file 1 [file jcm-15-08552-s001.zip › AF_AFL_Table_S7_JCM.pdf]

Supplementary Materials

Left Atrial Appendage Thrombus and Long-Term Clinical Outcomes in Anticoagulated Patients With Atrial Fibrillation and Atrial Flutter Referred for a Rhythm-Control Strategy: A Prospective Observational Cohort Study

Łukasz Turek, Marcin Sadowski, Łukasz Zandecki, Jacek Kurzawski, Marianna Janion

Table S7. Time-specific adjusted hazard ratios for covariates with time-varying coefficients in the extended Cox models.

Outcome	Covariate	1 year	3 years	5 years	8 years
Cardiovascular death	History of HF	3.208 (1.089–9.450)	1.342 (0.780–2.308)	0.895 (0.511–1.568)	0.616 (0.289–1.313)
First HF hospitalization	Previous stroke/TIA/systemic thromboembolism	1.117 (0.493–2.532)	2.163 (1.128–4.148)	2.941 (1.381–6.263)	3.901 (1.548–9.831)
First HF hospitalization	History of HF	0.933 (0.517–1.684)	0.647 (0.354–1.184)	0.546 (0.267–1.117)	0.467 (0.199–1.095)
First HF hospitalization	LVEF (per 1% increase)	0.973 (0.954–0.991)	0.980 (0.957–1.003)	0.983 (0.957–1.010)	0.987 (0.957–1.018)

Note: Values are adjusted HR (95% CI). Estimates were evaluated at 365.25, 1095.75, 1826.25, and 2922 days after index TEE, respectively. For each time-varying covariate X, $HR(t) = \exp[\beta X + \gamma X \times \ln(t)]$, with t measured in days. Confidence intervals were derived from the fitted coefficient covariance matrix: $Var[\beta X + \gamma X \ln(t)] = Var(\beta X) + 2 \ln(t) Cov(\beta X, \gamma X) + [\ln(t)]^2 Var(\gamma X)$.

Interaction P values (from the underlying models): history of HF for cardiovascular death, $p = 0.03$; previous stroke/TIA/systemic thromboembolism for first HF hospitalization, $p = 0.04$; history of HF for first HF hospitalization, $p = 0.11$; LVEF for first HF hospitalization, $p = 0.21$. The latter two interactions were not statistically significant. The LVEF HR is per one percentage-point increase. All estimates are model-based associations and are not causal effects; caution is warranted at later follow-up times and for wide confidence intervals.

Abbreviations: CI, confidence interval; HF, heart failure; HR, hazard ratio; LVEF, left ventricular ejection fraction; TEE, transesophageal echocardiography; TIA, transient ischemic attack.
