# Supplementary material for: Left Atrial Appendage Thrombus and Long-Term Clinical Outcomes in Anticoagulated Patients with Atrial Fibrillation and Atrial Flutter Referred for a Rhythm-Control Strategy: A Prospective Observational Cohort Study
Source: J Clin Med. 2026 Nov 3;15(21):8552. doi: 10.3390/jcm15218552 (PMC13763755; doi:10.3390/jcm15218552)
Supplement: Supplementary file 1 [file jcm-15-08552-s001.zip › AF_AFL_Table_S8_JCM.pdf]

# Supplementary Materials

Left Atrial Appendage Thrombus and Long-Term Clinical Outcomes in Anticoagulated Patients With Atrial Fibrillation and Atrial Flutter Referred for a Rhythm-Control Strategy: A Prospective Observational Cohort Study

Łukasz Turek, Marcin Sadowski, Łukasz Zandecki, Jacek Kurzawski, Marianna Janion

**Table S8.** Competing-risk sensitivity analyses for cardiovascular death and first HF hospitalization (AFL versus AF).

| Endpoint and model | Patients | Events | Competing deaths | Adjusted HR or sHR (95% CI) | *p* value |
|---|---|---|---|---|---|
| First HF hospitalization – Primary extended cause-specific Cox | 656 | 91 | 75 | aHR 0.69 (0.37–1.27) | 0.23 |
| First HF hospitalization – Cause-specific Cox, fixed baseline coefficients | 656 | 91 | 75 | aHR 0.68 (0.37–1.27) | 0.23 |
| First HF hospitalization – Fine–Gray, HF-first convention | 656 | 91 | 75 | sHR 0.70 (0.38–1.30) | 0.26 |
| First HF hospitalization – Fine–Gray, death-first convention | 656 | 89 | 77 | sHR 0.71 (0.38–1.33) | 0.29 |
| Cardiovascular death – Primary extended cause-specific Cox | 656 | 96 | 15 | aHR 1.09 (0.64–1.85) | 0.75 |
| Cardiovascular death – Cause-specific Cox, fixed baseline coefficients | 656 | 96 | 15 | aHR 1.07 (0.63–1.83) | 0.79 |
| Cardiovascular death – Fine–Gray | 656 | 96 | 15 | sHR 1.07 (0.63–1.83) | 0.79 |

Note: For cardiovascular death, the competing event was non-cardiovascular death (96 cardiovascular deaths, 15 competing deaths, 545 censored). The primary extended Cox model included 10 baseline covariates and one time-varying coefficient; the competing-risk sensitivity models included the same 10 baseline covariates with fixed coefficients. For first HF hospitalization, the competing event was death from any cause before the first HF hospitalization. The primary extended Cox model included 14 baseline covariates and three time-varying coefficients; corresponding sensitivity models used the same 14 baseline covariates with fixed coefficients.

For first HF hospitalization, two patients had hospitalization and death on the same recorded day. The primary competing-risk analysis prioritized HF hospitalization (91 HF events, 75 competing deaths, 490 censored), whereas the alternative prioritized death (89 HF events, 77 competing deaths, 490 censored). Of 111 deaths overall, 34 occurred after a recorded HF hospitalization, 75 occurred before hospitalization, and two were same-day events. Cause-specific HRs and subdistribution HRs represent different estimands and should not be directly interpreted as risk ratios. These observational analyses do not establish equivalence or causality.

Abbreviations: AF, atrial fibrillation; AFL, atrial flutter; aHR, adjusted cause-specific hazard ratio; CI, confidence interval; HF, heart failure; HR, hazard ratio; sHR, subdistribution hazard ratio.
