# Supplementary material for: Left Atrial Appendage Thrombus and Long-Term Clinical Outcomes in Anticoagulated Patients with Atrial Fibrillation and Atrial Flutter Referred for a Rhythm-Control Strategy: A Prospective Observational Cohort Study
Source: J Clin Med. 2026 Nov 3;15(21):8552. doi: 10.3390/jcm15218552 (PMC13763755; doi:10.3390/jcm15218552)
Supplement: Supplementary file 1 [file jcm-15-08552-s001.zip › AF_AFL_Table_S9_JCM.pdf]

Supplementary Materials

Left Atrial Appendage Thrombus and Long-Term Clinical Outcomes in Anticoagulated Patients With Atrial Fibrillation and Atrial Flutter Referred for a Rhythm-Control Strategy: A Prospective Observational Cohort Study

Łukasz Turek, Marcin Sadowski, Łukasz Zandecki, Jacek Kurzawski, Marianna Janion

Table S9. Variance inflation factors (VIFs) for baseline covariates in the primary survival models.

Baseline covariate	CV death: primary model	CV death: excluding CHA_2DS_2-VA	HF hospitalization: primary model	HF hospitalization: excluding CHA_2DS_2-VA
Index arrhythmia type (AFL vs AF)	1.07	1.05	1.12	1.12
Left atrial appendage thrombus	1.14	1.13	1.14	1.14
Age	1.91	1.28	2.59	1.29
CHA_2DS_2-VA	2.32	–	5.39	–
Heart rate	–	–	1.19	1.19
eGFR (MDRD)	1.24	1.23	1.27	1.27
Left atrial diameter	1.17	1.16	1.20	1.19
LVEF	1.38	1.38	1.49	1.49
Previous stroke/TIA/systemic thromboembolism	–	–	1.73	1.02
History of heart failure	1.48	1.17	1.84	1.20
Diabetes mellitus	–	–	1.59	1.03
Previous myocardial infarction	1.10	1.04	1.20	1.05
Peripheral artery disease	1.04	1.01	1.07	1.02
VKA on admission	–	–	1.06	1.06
Maximum VIF	2.32	1.38	5.39	1.49

Note: VIFs were calculated by regressing each baseline covariate on the other baseline covariates in the respective model ($VIF = 1/[1 - R^2]$). The primary cardiovascular-death model included 10 baseline covariates; the primary HF-hospitalization model included 14. The reduced models exclude CHA_2DS_2-VA only. The VIF calculation does not incorporate covariate-by-log(time) interactions from the extended Cox models; therefore, it does not fully characterize collinearity of the time-dependent model design. These VIFs describe predictor redundancy, not model discrimination or coefficient precision. A dash denotes a covariate absent from that model.

Abbreviations: AF, atrial fibrillation; AFL, atrial flutter; CHA_2DS_2-VA, clinical thromboembolic risk score without the sex category; CV, cardiovascular; eGFR, estimated glomerular filtration rate; HF, heart failure; LAAT, left atrial appendage thrombus; LVEF, left ventricular ejection fraction; MDRD, Modification of Diet in Renal Disease; TIA, transient ischemic attack; VIF, variance inflation factor; VKA, vitamin K antagonist.
